# Neurotoxicity of Benzotriazole Ultraviolet Stabilizers in Teleost Fishes: A Review

**DOI:** 10.3390/toxics12020125

**Published:** 2024-02-02

**Authors:** Mengli Li, Emma Ivantsova, Xuefang Liang, Christopher J. Martyniuk

**Affiliations:** 1 Inner Mongolia Key Laboratory of Environmental Pollution Control & Waste Resource Reuse, School of Ecology and Environment, Inner Mongolia University, Hohhot 010021, China; limengli0718@126.com (M.L.); liangxf@imu.edu.cn (X.L.); 2 Department of Physiological Sciences and Center for Environmental and Human Toxicology, University of Florida Genetics Institute, Interdisciplinary Program in Biomedical Sciences Neuroscience, College of Veterinary Medicine, University of Florida, Gainesville, FL 32611, USA; eivantsova@ufl.edu

**Keywords:** plasticizers, central nervous system, aquatic toxicology, BUVS, behavior, plastic additives

## Abstract

Plastic additives that maintain integrity have been extensively studied for potential toxicity to fish; however, chemicals that protect polymers from (artificial) UV degradation are less studied. Benzotriazole UV stabilizers (BUVSs) are the most widely used UV stabilizers in plastics and are often used in sunscreens, cosmetics, paint, and food packaging. BUVSs can negatively affect aquatic wildlife when released into the environment via plastic degradation. In this review, we summarize the distribution of BUVSs globally and discuss neurotoxicological endpoints measured in fish to understand how these plastic additives can affect the neurological health of teleost fishes. BUVSs have been detected in aquatic environments at concentrations ranging from 0.05 up to 99,200 ng/L. Studies show that BUVSs affect behavioral responses and acetylcholinesterase activity, indicators of neurotoxicity. Our computational analysis using transcriptome data suggests certain pathways associated with neurodegeneration are responsive to exposure to BUVSs, like “Complement Activation in Alzheimer’s Disease”. Based on our review, we identify some research needs for future investigations: (1) molecular studies in the central nervous system to define precise mechanisms of neurotoxicity; (2) a wider range of tests for assessing aberrant behaviors given that BUVSs can affect the activity of larval zebrafish; and (3) histopathology of the nervous system to accompany biochemical analyses. These data are expected to enhance understanding of the neurotoxicity potential of benzotriazoles and other plastic additives.

## 1. Introduction: Ultraviolet Stabilizers, Non-Negligible Additives in Plastics 

Microplastic pollution has received intensifying attention as an emerging worldwide environmental issue. One of the most important problems with microplastics is the leaching of harmful additives [1]. During plastic production, chemical additives are often added to improve the performance, functionality, and ageing properties of the polymer. Plastics often contain four types of plastic additives: plasticizers, flame retardants, light stabilizers, and antioxidants. Amongst these additives, plasticizers and flame retardants are the most frequently added, at a proportion of >70% to products. Plasticizers are functional additives that improve plasticity, flexibility, processability, and durability [2]. They are mostly used in polyvinyl chloride (PVC) products, but are also used in wires, cables, coatings, product packaging, cosmetics, pharmaceuticals, and medical devices [3,4,5,6]. Currently, there are over 500 types of plasticizers produced industrially. To protect plastics and plasticizers, other stabilizing chemicals are embedded in polymer resins to delay the overall oxidative degradation of plastics if/when exposed to ultraviolet (UV) light [7]. Although they only account for 0.05–3% of the number of additives used in plastic manufacturing, their ubiquitous detection in the environment and biota, as well as potential toxicity to aquatic organisms, has led to increased concern regarding their aquatic ecosystem presence. 

**Figure 1:**
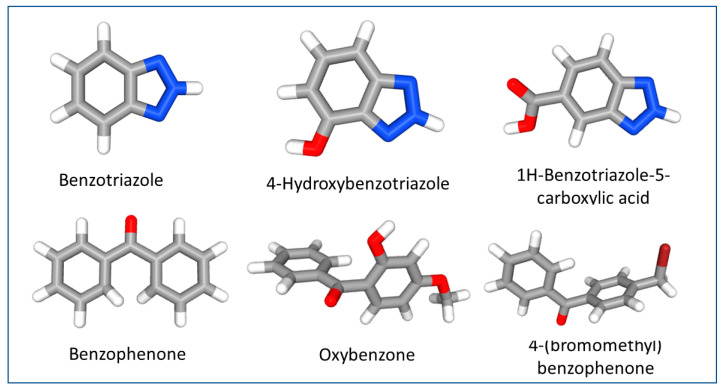
A 3D depiction of examples of different benzotriazoles, benzophenones, and their derivatives. Red indicates the position of oxygen, and blue indicates the position of nitrogen. Images extracted from the National Center for Biotechnology Information (2023). PubChem Compound Summary. Images retrieved 1 March 2023.

UV stabilizers are a group of substances that protect polymers from degradation caused by sunlight or other artificial UV light. Of these, benzotriazole derivatives are the most widely used UV stabilizers in plastics [8]. Benzotriazole UV stabilizers (BUVSs) share a common 2-hydroxyphenyl benzotriazole structure, and the derivatives are classified by various alkyl substitutions on the phenol ring (Figure 1). BUVSs can absorb the full spectrum of UV light, including UV-A (320–400 nm) and UV-B light (280–320 nm), to prevent light-induced yellowing and degradation of products [9]. Due to these unique properties, benzotriazole UV stabilizers (BUVSs) are often used in sunscreen, cosmetics, and other personal care products [10,11,12], as well as in plastics, paints, and food packaging [13,14]. BUVSs have been added to the list of high-production-volume (HPV) chemicals [15].

## 2. Objectives of the Review

The objectives of this review were to (1) summarize the distribution of BUVSs on a global scale and (2) review studies in fish to better understand how these plastic additives may affect the neurological health of aquatic species. While exposure to BUVSs can have a range of biological effects in fish, including oxidative stress, reproductive disruption, and immunotoxicity [16,17,18], we elect to focus here on neurotoxicity, as several studies now report that microplastics negatively affect the central nervous system of fish [19]. Evidence shows that BUVSs can affect common biomarkers of neurotoxicity, including acetylcholinesterase activity and neurotransmitter concentrations in fish. 

**Figure 2:**
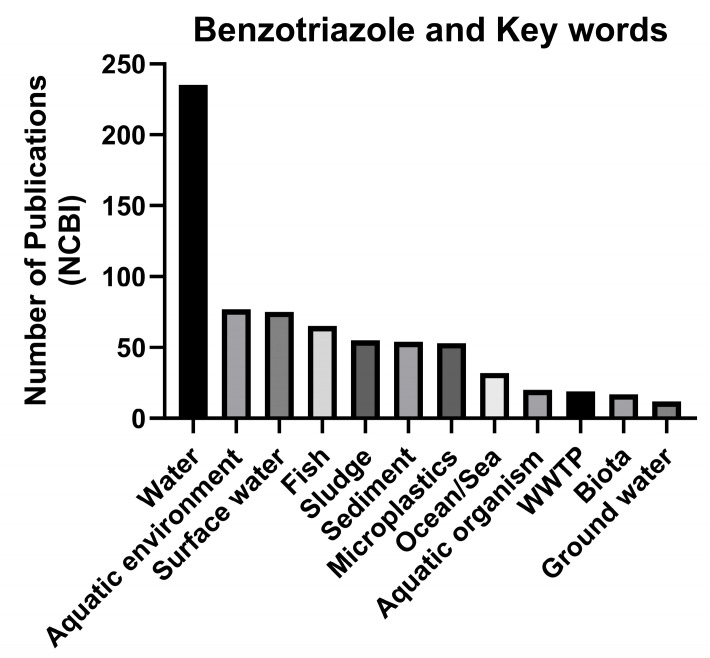
The number of studies corresponding to keywords searched.

A literature search was conducted on the Web of Science (www.webofknowledge.com) and ScienceDirect (https://www.sciencedirect.com) accessed on 1 August 2023 using the keywords [benzotriazole UV stabilizers or benzotriazole UV filters + aquatic environment or water or sludge or sediment]. A total of 365 papers were collected and surveyed for information regarding environmental concentrations in sediment, soil, and water. Figure 2 displays the number of studies corresponding to each keyword. Of these, 64 toxicological studies on the neurotoxicity of benzotriazole UV stabilizers and benzophenone UV filters were identified using the keywords [BUVSs + neurotoxicity OR nervous system OR locomotion behavior OR anxiety]. Of these studies, there were 11 reports on neurotoxic endpoints (molecular, biochemical, and behavioral) of BUVSs in fish. Data from these studies were synthesized to generate a more complete understanding of how BUVSs may affect neural-related endpoints and identify potential knowledge gaps in the literature. 

## 3. Occurrence of Ultraviolet Stabilizers in Aquatic Environments 

**Figure 3:**
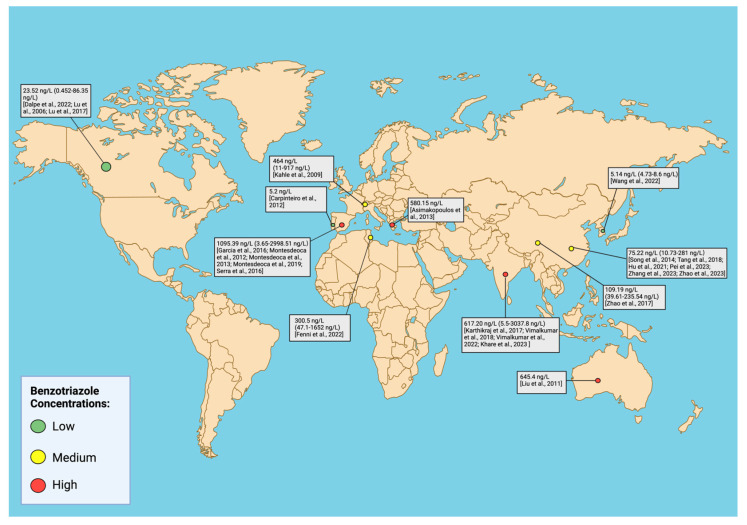
A global map summarizing benzotriazole and benzophenone concentrations in surface water and wastewater. Supplemental Table S2 categorizes concentrations by country/region [14,20,25,26,36,37,38,39,40,41,42,43,44,45,46,47,48,49,50,51,52,53,54,55,56]. Created with BioRender.com.

BUVSs can be deposited in aquatic ecosystems via wastewater treatment plant discharges [20], runoff [21], landfills [22], and plastic debris [8,23,24]. These chemicals have been detected in environmental samples such as surface water [14], wastewater [25], seawater [26], sediment [14,27], and sewage sludge [22,28]. Supplemental Table S1 summarizes data for global benzotriazole concentrations. Limited studies examine environmental concentrations in Africa, Australia, and South America, as only four studies in total have reported benzotriazole concentrations at these locations. In North America, multiple studies evaluate concentrations of BUVSs in the USA or Canada; however, most studies available that report on benzotriazole concentrations are in Europe and Asia, in countries such as Spain, China, and India. Briefly, BUVSs (e.g., UV-P, UV-320, UV-326, UV-327, UV-328, and UV-329) were detected in water from rivers in central India at concentrations up to 6.79 ng/L [14], while UV-360 was detected in Gran Canaria seawater at concentrations ranging from 41.12 to 544.9 ng/L [26]. BUVSs (e.g., UV-P, UV-329, UV-326, UV-328, UV-327, UV-571, and UV-360) were present in Gran Canaria (Spain) wastewater at concentrations up to 83.3 ng/L (UV-326) [25]. Additionally, they were detected at concentrations ranging from 0.3 to 320 ng/g dry weight [27] and from 44 to 2362 ng/g dry weight [22] in sediment and sewage sludge, respectively. Studies have also detected BUVSs in human breast milk [29], urine [30], indoor dust [31], and in the tissues of fish, birds, and other aquatic organisms [22,32,33,34]. Due to their high lipophilicity, BUVSs show a high propensity to bioaccumulate in aquatic organisms [35]. For instance, reports indicate that BUVSs can reach up to 450 ng/g lipid weight in mussels analyzed across 10 countries [34]. Figure 3 provides an overview of BUVS concentration globally.

## 4. Neurotoxicity of Ultraviolet Stabilizers in Fish

The health of the central nervous system is critical to all physiological processes in an individual. Unfortunately, there is a plethora of environmental contaminants that can induce neurotoxicity in fish, including pesticides, industrial chemicals, and plastic debris. Researchers have utilized several cellular, biochemical, and molecular endpoints for evaluating the neurotoxicity potential of chemicals, including enzyme activity like acetylcholinesterase or molecular indicators of neuronal or glial damage (e.g., beta-tubulin, microtubule-associated protein tau, glial fibrillary acidic protein). In the following section below, we discuss studies that support or refute the hypothesis that benzotriazoles induce neurotoxicity in fish. Zebrafish were of focus as they share many genes and neurotransmitters, as well as a similar central nervous system structure, with humans, which make them valuable models of neurotoxicity. Table 1 provides a summary of the neurotoxic endpoints assessed and the outcomes of the exposure experiments in fish. However, we also point out that there are additional studies on marine species, mainly bivalves, that address the toxicity of BUVSs. We have included this information in Supplemental Table S3 as a resource to guide future studies in marine invertebrates. 

### 4.1. Molecular and Biochemical Indicators of Neurotoxicity

There have been several studies reporting the neurotoxic effects of BUVSs at the cellular and molecular level. Song et al. [65] exposed larval zebrafish (*Danio rerio*) to 0.8, 1, 1.2, 1.6, or 2.4 µg/mL and adult zebrafish to 1, 10, 100, or 1000 µg/L benzophenone-1 (BP-1) and reported that BP-1 decreased the number of dopaminergic neurons in the midbrain and induced the loss of neurons in the central nervous system (midbrain and thalamus) in a concentration-dependent manner. Zebrafish exposed to 2.4 µg/mL also lost a significant number of neurons in their tails. Neurodevelopment-related genes in larvae were also measured in the study following 6 days of exposure. Following exposure to 1.6 and 2.4 µg/mL, brain-derived neurotrophic factor (*bdnf*) and myelin basic protein a (*mbpa*) decreased and increased in expression, respectively. Additionally, pro-opiomelanocortin (*pomc*) expression was reduced in fish exposed to 0.8 and 1.0 µg/mL, and glial fibrillary acidic protein (*gfap*) expression was reduced in fish exposed to between 1.0 and 2.4 µg/mL [65]. Additional studies support the hypothesis that BUVSs induce oxidative stress and neuroinflammation in fish. For example, Sun et al. [64] exposed zebrafish embryos to 10 or 100 µg/L benzophenone-3 (BP-3) or titanium dioxide nanoparticles (nano-TiO_2_) for 1 day, either separately or in a mixture. Single and co-exposure to BP-3 induced apoptosis, inhibited axonal growth, and generated reactive oxygen species (ROS). The immune system also appears to be a target for toxicity, indicating the potential for neuroinflammation. In another study, Zhang et al. [57] exposed zebrafish embryos to two common benzotriazole UV stabilizers, UV-234 and UV-326, at concentrations of 1, 10, and 100 µg/L for 7 days. In the study, pro-inflammatory gene expression was analyzed, and it was found that the two stabilizers had varying effects on the organism, suggesting differences in mechanisms of toxicity. In fish exposed to 100 µg/L UV-326, interleukin-1 beta (*il1β*) and interleukin 6 (*il6*) were significantly increased; however, *il1β* was significantly decreased in fish exposed to 100 µg/L UV-234. Tumor necrosis factor α (*tnfα*), a pro-inflammatory cytokine, was significantly decreased following both stabilizer treatments. Insulin-like growth factor 1 (*igf1*) and stromal cell-derived factor 1 (*sdf1a*) were inhibited 1.65- to 2.26-fold and 2.15- to 2.19-fold, respectively, following UV-234 exposure, which indicates damage to cell differentiation and regeneration function. In fish exposed to UV-326, igf1 decreased with 10 µg/L, but fibroblast growth factor 2 (*fgf2*), matrix metalloproteinase 9 (*mmp9*), and *sdf1a* increased with exposure to 1 or 100 μg/L UV-326, indicating the initiation of nerve cell repair following an inflammatory reaction [57]. Furthermore, at the biochemical level, exposure to BP-3 for 3 days decreased acetylcholinesterase (AChE) activity and increased AChE gene expression in a concentration-dependent manner in zebrafish [62]. Taken together, oxidative stress, neuroinflammation, and effects on AchE are notable mechanisms of toxicity for BUVSs. 

### 4.2. Behavioral Indicators of Neurotoxicity

Behavioral assays have been utilized to identify interactions of ultraviolet stabilizers with the nervous system. Behavioral endpoints measured that provide sensitive indicators for neuroactivity of BUVSs in fish have included spontaneous tail coiling (STC), alternating light- and dark-induced locomotor response (LMR-L/D), and social behaviors such as shoaling, and mirror response (Table 1). To date, several toxicological studies have reported on ultraviolet filters, including benzophenone derivatives like BP-1 and BP-3 (BP-3 being the most studied) [66]. These studies have been conducted on both larval and adult fish. Tao and colleagues [59] exposed zebrafish embryos to 1, 10, or 100 µg/L BP-3 for up to 4 days. Axon length was decreased in 27 hpf larvae, and cell apoptosis in the head region increased in all treated embryos. Concerning motor and social behavior analysis, only embryos exposed to 10 µg/L were analyzed. Response rates in 27 hpf larvae were reduced, but this effect was not observed in 48 hpf larvae. Swimming distance and speed were increased at 5 dpf, the nearest neighbor distance and the inter-individual distance increased significantly at 11 dpf, and the mean number of times fish butted/bit or spent around the mirror during the mirror response test were significantly decreased at 12 dpf. In another study, larval zebrafish exposed to between 0.8 and 2.4 µg/mL BP-1 for 6 days showed reduced velocity when exposed to the highest tested concentration [59]. Sun et al. [64] exposed zebrafish embryos separately or in a mixture to 10 or 100 µg/L BP-3 or nano-TiO_2_ for 1 day. At 24 hpf, both single and co-exposure significantly increased spontaneous movement, and, at 30 hpf, co-exposure of the chemicals caused the touch response rate to decrease.

Adult fish behavioral responses have also been investigated following BUVSs exposure. Moreira and Luchiari [57] analyzed the response of adult zebrafish exposed to 10, 100, or 1000 µg/L BP-3 following several administered tests (i.e., T-maze, shoal preference, mirror test, novel tank test). Decreased exploration and interaction of the novel arm in the T-maze and with the shoal preference test were observed in fish, along with fewer mirror image interactions, decreased anxiety-like behavior, and decreased locomotion. Furthermore, Bai et al. [60] exposed adult zebrafish to 10 µg/L BP-3 for 150 days and analyzed behavioral alterations and cognitive deficits. Following a social preference test, fish were observed to have reduced prosocial behaviors. Additionally, biting behavior in females was significantly reduced. Both male and female fish were found to display impaired learning and memory and neurogenesis inhibition [60]. In female fish, brain weight and dopamine levels were reduced, and the number of apoptotic cells in their telencephalon was increased. In another study using a T-maze test in adult zebrafish exposed to BP-1, impaired learning and memory ability were observed as retention time decreased and latency to deep water increased in a concentration-dependent manner [25]. The total swimming distance in adults also decreased in a concentration-dependent manner, and the swimming duration at the bottom of the tank increased, indicating that BP-1 decreases exploratory behavior at the top of the tank (i.e., increases anxiety). 

Conversely, behavioral studies on UV stabilizers are limited. As a derivative of the suspected neurotoxin benzotriazole [67], many BUVS congeners are reported to disrupt swimming behavior in early-stage zebrafish. Liang et al. [58] exposed zebrafish embryos to 0.01, 0.1, or 1.0 µM UV-234 or UV-320 for 6 days and conducted a light/dark locomotor response assay. There were subtle behavioral responses that were chemically dependent. Concerning fish in the UV-234 exposure group, larvae pre-adapted to darkness and exhibited decreased locomotor activity during the beginning of the assay. Zebrafish larvae exposed to 0.1 and 1 µM exhibited a decrease in overall distance moved, whereas fish exposed to 0.01 µM exhibited an increase in distance moved. Concerning fish in the UV-320 exposure group, larvae pre-adapted to darkness at the beginning of the assay and exhibited decreased activity. Fish pre-adapted to darkness and exposed to 1 µM initially exhibited increased activity and distance moved but exhibited a decrease in distance moved near the end of the exposure. Additionally, fish treated with 0.1 µM exhibited increased distance moved during the light phase of the assay. Thus, it appears that the type of BUVS and its concentration can induce variable responses in zebrafish larvae. In another study, Zhang et al. [57] exposed zebrafish embryos to UV-234 and UV-326 at 1, 10, and 100 µg/L for 7 days. Concerning behavioral alterations following a light/dark locomotor response assay, both compounds were found to induce hyperactivity during the dark cycles of the assay, where acceleration, mobile activity, and swimming distance were increased. Additionally, spontaneous tail coiling was inhibited 2.08–6.25-fold from 28–29 hpf following exposure to both stabilizers. Noteworthy is that these behavioral responses corresponded to the activity of AChE, which was elevated in fish exposed to 10 and 100 µg/L UV-234 and UV-326. Taken together, aberrant behavior has been associated with BUVS-induced apoptosis and ROS generation, which subsequently influence neuroinflammation and cell regeneration processes [57]. Similar molecular events have also been observed in Asian clams (*Corbicula fluminea*) [68], where impairment of the oxidative defense system induced by BUVSs triggered an increase in the neurotransmitter acetylcholine, causing accelerated filtration and tissue damage, suggesting that the neurotoxic effects of BUVSs may not be limited to aquatic vertebrates. 

## 5. Biomarkers of Toxicity: Neurotoxic Indicators?

To uncover molecular responses underlying benzotriazole and benzotriazole derivatives, we extracted molecular interactions for further investigation using Pathway Studio (V12) as per our previous methods [69,70]. Genes differentially affected by benzotriazole were extracted from the comparative toxicogenomics database (CTD) [71]. We were able to identify cell processes, cell components, clinical parameters, proteins, and other functional classes with direct connections to benzotriazole. These interactions have been implicated in experiments investigating benzotriazoles. 

**Figure 4:**
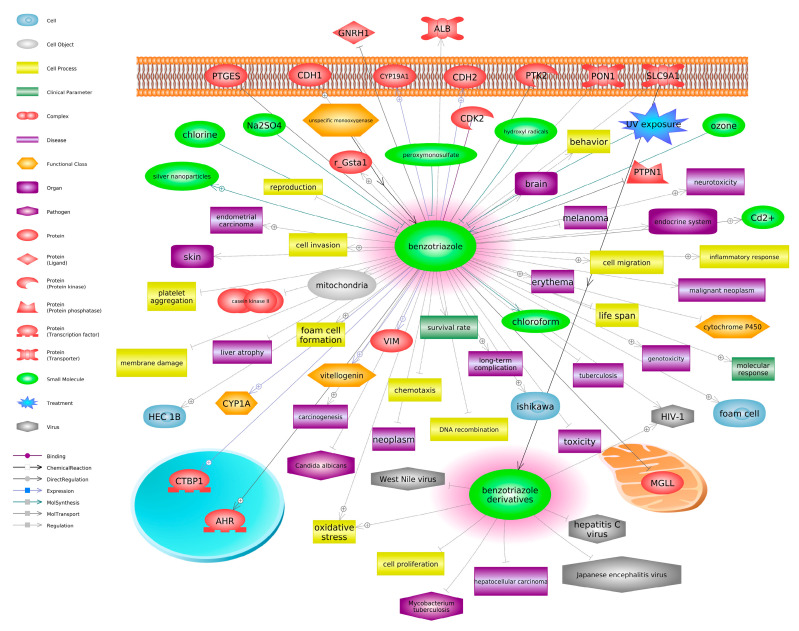
Functional classes and proteins associated with benzotriazole [Pathway Studio (Elsevier)]. Abbreviations for the figure are presented in Supplemental Data S1.

There were over 50 entities that were associated with benzotriazole exposure. Several small molecules were linked to benzotriazoles (i.e., ozone, chloroform, Cd^2+^, and chlorine), as well as several viral entities (i.e., hepatitis C, HIV-1) and pathogens (i.e., *Candida albicans* and *Mycobacterium tuberculosis*) (Figure 4). Other notable entities associated with benzotriazoles included chemotaxis, oxidative stress, DNA recombination, mitochondria, genotoxicity, endometrial carcinoma, and liver atrophy. Transcripts or proteins responsive to benzotriazoles included PTPN1 (protein tyrosine phosphatase non-receptor type 1), PTGES (prostaglandin E synthase), GNRH1 (gonadotropin releasing hormone 1), AHR (aryl hydrocarbon receptor), CDH1 (cadherin 1), and CDK2 (cyclin dependent kinase 2). Many of these proteins have been implemented in neurodegenerative diseases and are linked to protective effects for neural toxicity. For example, paraoxonase-1 can neutralize environmental toxicants to protect lipids against peroxidation. Consequently, the deterioration and absence of paraoxonase-1 removes mechanisms responsible for breaking down neurotoxic compounds, which can increase a species’ vulnerability to neurotoxic consequences [72,73]. CDH1 is a transmembrane protein involved in mediating cell-to-cell adhesion, thus playing a significant role in tissue homeostasis, including in the blood–brain barrier. Transcription of cadherin-1 has been found to increase in the presence of neuronal degeneration and astrocyte swelling following silver nanoparticle exposure, which demonstrates its protective effects against neurotoxicity [74]. CDK2 positively regulates cell cycle progression and has been found to be essential in mice for the regeneration and growth of neural progenitor cells [75]. Disruption to the regeneration and proliferation of progenitor cells can alter the communication abilities of the central nervous system. Lastly, AHR connects environmental chemical inputs to adaptive reactions, including metabolic processes and immunological responses. Disruption of the receptor, specifically its inactivation, has been found to negatively regulate transcriptional responses related to microglial activation and neurotoxic monocyte recruitment [76]. 

In addition, gene set enrichment revealed pathways like “Complement Activation in Alzheimer’s Disease”, “MPB-Related Complement Cascade Activation”, and “Positive Acute Phase Protein Synthesis” were associated with BUVSs exposure (Table 2). Some studies report upregulated AChE activity following UV-234 and UV-326 exposure [57], which can result in increased amyloid-beta deposition, thus contributing to Alzheimer’s disease. Such targets can be further investigated for their role in BUVS-induced neurotoxicity. 

Evidence for neurotoxicity was also noted in the interaction network with cell invasion, membrane damage, and behavior (Figure 4); thus, evidence suggests the neural and immune systems are relevant targets for adverse outcomes related to benzotriazole exposure. Such relationships have yet to be investigated in fish, presenting an opportunity to investigate novel mechanisms of action for this chemical class. Pathways responsive to BUVSs retrieved from the Comparative Toxicogenomics Database [71] included glutathione conjugation, glutathione metabolism, drug metabolism, cytochrome P450, and regulation of Toll-like receptors (TLRs) by endogenous ligands. Benzotriazoles have been shown to affect these processes in animals. He et al. [77] exposed marine medaka (*Oryzias melastigma*) to 0.01–1.0 mg/L benzotriazole for 35 days and observed decreased expression of cytochrome P450 1A1 (*cyp1A1*) in the liver and intestines of fish, which is responsible for steroidal hormone metabolism and immune response enhancement. Another study conducted by Hemalatha et al. [61] found that BUV-328 elevated glutathione S-transferase in adult zebrafish exposed to 0.01–1 mg/L for up to 42 days, denoting the metabolization of lipid peroxides and the biotransformation of toxic compounds. Additionally, zebrafish embryos exposed to 15.8–690 μg/L 2-(2-Hydroxy-5-methylphenyl) benzotriazole (UV-P) exhibited a dose-related induction of glutathione-S-transferase, whereas the opposite effect was observed in fish exposed to 7.5–84.3 μg/L UV-326 for 6 days [78]; these data suggest that benzotriazole and its derivatives have varying impacts on detoxification processes, which is accomplished by the catalytic conjugation of glutathione. The computational analysis also points to glutathione depletion, oxidative stress, and neuro-inflammation as underlying neurological dysfunction and behavioral abnormalities following BUVS exposure.

## 6. Conclusions 

**Figure 5:**
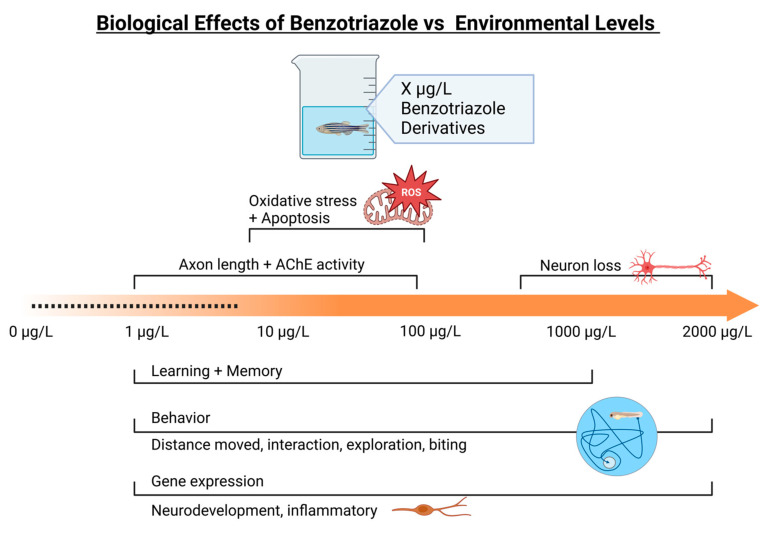
The neurotoxic effects of benzotriazole in fish compared to measured environmental concentrations (dashed line indicates environmental levels). Created with BioRender.com.

Studies report low acute toxicity of BUVSs to fish; however, the ecotoxicological risk to species is not fully elucidated. Benzotriazoles and their derivatives are highly stable in soil/water due to their physiochemical properties, which allow them to persist in the environment. In soil, benzotriazole has been reported to have a half-life of 180 days at 20 °C, and, in freshwater, its half-life is 831 days at 12 °C (https://echa.europa.eu/registration-dossier/-/registered-dossier/14234/5/3/4) (accessed on 1 August 2023). Though most studies report benzotriazoles and their derivatives in ng/L, with the compound’s resistance to biodegradation and its continued utilization, it can accumulate in the environment, thus posing threats to aquatic organisms. We point out deficiencies in the literature currently related to the neurotoxicity of BUVSs. These knowledge gaps include the following:

(1) In-depth mechanistic studies on the central nervous system of zebrafish are needed to address neurotoxicity. Validation of specific neurotoxicity pathways relevant for BUVS exposure is needed.
(2) Broader scope of behavioral assays related to the dopaminergic systems, such as anxiety-related and fear-related behaviors, given that the exploration of novel tank environments by fish is altered with exposures.
(3) Histopathology of the central nervous system is needed following exposure to these chemicals, given evidence for neuronal damage, apoptosis, and neurodegeneration.
(4) Ecologically important species would broaden the scope and environmental relevance of laboratory-based studies, as most studies are conducted using zebrafish. Nevertheless, the zebrafish model has proven useful for developmental toxicity studies for plasticizers and has improved our understanding of toxicity mechanisms in fish.
(5) Based on our review, several studies report neurological responses above environmental levels (Figure 5), although there are experimental data that correspond to environmental levels.

One final point to make is that most concentration data are collected in non-marine environments. Benzotriazoles and benzophenone can also exert toxic effects on marine organisms like sea urchin larvae and coral [79,80]. However, to our knowledge, the studies on adverse effects on marine fish are limited. Research efforts in these areas are expected to fill knowledge gaps regarding the effects of UV stabilizers in the central nervous system of fish and aquatic species in general. Such approaches will facilitate risk assessments and identify safe environmental levels for this ubiquitous class of chemicals. 

## Figures and Tables

**Table 1:** Summary of neurotoxic endpoints and outcomes in zebrafish following chemical exposure.

Chemicals	Dose	Life Stage	Exposure Period	Endpoint	Results	Reference
UV-234,UV-326	1, 10, 100µg/L	Embryos	7d	AchE activity	Upregulated at 10 and 100 μg/L	Zhang et al., 2023 [57]
				Locomotor response	Both compounds induced hyperactivity in the dark cycle via swimming distance, acceleration, and mobile activity.	
				Neurotrophic factors	igf1 and sdf1a were inhibited 1.65- to 2.26-fold and 2.15- to 2.19-fold, respectively, with UV-234; mmp9, fgf2, and sdf1a increased with 1 and 100μg/L UV-326; igf1 decreased with 10 μg/L exposure UV-326	
				Spontaneous tail coiling (STC)	Inhibited 2.08–6.25-fold	
				Pro-inflammatory gene expression	*tnfα* decreased in all treatment. *il1β* decreased with 100 μg/L UV-234 and increased with 100 μg/ L UV-326; *il6* increased with 100 μg/ L UV-326	
UV-234, UV-320	0.01, 0.1, 1 µM	Embryos	6d	Locomotor response	UV-234 altered activity in both light/dark periods; Hyperactivity was induced in fish pre-adapted to darkness with 1 µM UV-320; 1 µM UV-320 increased distance moved in the dark phase; 0.1 µM UV-320 increased distance moved in the light phase	Liang et al., 2019 [58]
BP3	1, 10, 100 µg/L	Embryos and larvae	4d	Axonal Growth	Decreased relative axon length in 27 hpf larvae.	Tao et al., 2020 [59]
				Touch response	Decreased in 27 hpf larvae with 10 μg/L	
				Locomotor response	Increased swimming distance and average swimming speed in the dark period with 10 µg/L	
				Spontaneous movement	Increased frequency of bending at 21 hpf (10 and 100 μg/L) and 24 hpf (10 μg/L)	
				Social behaviors	Nearest neighbor distance and the inter-individual distance increased; Mean attacks and time spent in the mirror area decreased	
BP3	10 µg/L	Adults	150d	Social preference	Reduced prosocial behaviors	Bai et al., 2023 [60]
				Mirror biting test	Reduction of biting behavior in females	
				T–maze test	Impaired learning and memory regardless of sex	
				Body length, weight, brain weight, brain dopamine and acetylcholine	Reduced female brain weight and dopamine level	
				Cell proliferation in the telencephalon	Neurogenesis inhibited in the telencephalon	
				Cell apoptosis in the telencephalon	Apoptotic cells increased in the female telencephalon	
BP3	2 mg/L	Larvae	5d	Enteric neuron number and related gene expression	BP-3 could impede ENS zebrafish development via the MAPK/ERK signaling pathway	Hemalatha et al., 2020 [61]
BP3	1, 10 µg/L	Embryos	3d	AChE	Inhibited by both concentrations	Sandoval-Gío et al., 2021 [62]
BP3	10, 100, 1000 µg/L	Adults	15d	Novel tank test	Reduced locomotion and decreased anxiety-like behavior	Moreira et al., 2022 [63]
				Shoal preference	Reduced interaction and time near the shoal	
				Mirror test	Reduced interactions with the mirror image; thus, impairing proper aggressive response	
				T-maze	Reduced exploration of the novel arm; thus, jeopardizing the ability to retain information	
BP3, nano-Tio2	10 µg/L BP3; 100 µg/L nano-Tio2 (separately and combined)	Embryos	1d	Spontaneous movement	Increased in single and coexposure groups at 24 hpf	Sun et al., 2023 [64]
				Touch response	Decreased in co-exposure at 30 hpf	
				Axonal growth	Single and coexposure inhibited axonal growth, and induced apoptosis and ROS generation	
BP1	0.8, 1, 1.2, 1.6, 2.4 µg/mL	Larvae	4d	CNS	Abnormal brain structure and neuron loss	Song et al., 2022 [65]
				DA neurons	Decreased the number in the midbrain	
			6d	Locomotor capacity	Suppressed velocity and movement distance; altered expression of neurodevelopment related genes	
BP1	1, 10, 100, 1000 µg/L	Adults	14d	T-maze tests	Inhibited spatial working memory	
				Tank diving tests	Increase in proportion of bottom swimming duration/distance to total duration/distance, indicating a decrease of exploratory behavior	

**Table 2:** Gene set enrichment of genes regulated by benzotriazole (*p* < 0.001). The name of the pathway, the number (#) of entities in the pathway, and the overlap between regulated genes and those in the pathway. percent overlap, *p*-value, and hit type are included in the table.

Name	Expanded # of Entities	Overlap	Percent Overlap	Hit Type
Complement Activation in Alzheimer’s Disease	36	10	27	Disease
MPB-Related Complement Cascade Activation	39	10	25	Disease
Positive Acute Phase Proteins Synthesis	609	20	3	Biological Process
Trophoblast Damage in Infertility (Hypothesis)	61	10	16	Disease
CD46/CD55/CD59 Inhibit Complement Mediated Lysis of Cancer Cells	50	10	20	Pathological Process
Complement Activation in Glomerulonephritis	63	10	15	Disease
Complement System Defects in Systemic Lupus Erythematosis	73	10	13	Disease
Complement Activation by Lectin	68	10	14	Biological Process
Extraocular Muscles Weakness in Myasthenia Gravis	84	10	11	Disease
Complement Classical Pathway	71	10	14	Biological Process

## Data Availability

The original data presented in the study are included in the article/Supplementary Materials.

## Supplementary Materials

The following supporting information can be downloaded at: https://www.mdpi.com/article/10.3390/toxics12020125/s1, Supplemental Table S1: Occurrence of ultraviolet stabilizers in aquatic environments. Supplemental Table S2: Occurrence of ultraviolet stabilizers in aquatic environments. Supplemental Table S3: presents data for marine invertebrates. Supplemental Data S1: contains all abbreviations for the pathway in Figure 4. References [81,82,83,84,85,86,87,88,89,90,91,92,93,94,95,96,97,98] are cited in the supplementary materials.
