# Supplementary material for: Neurotoxicity of Benzotriazole Ultraviolet Stabilizers in Teleost Fishes: A Review
Source: Toxics. 2024 Feb 2;12(2):125. doi: 10.3390/toxics12020125 (PMC10891865; doi:10.3390/toxics12020125)
Supplement: Supplementary file 1 [file toxics-12-00125-s001.zip › Table S1.pdf]

Table S1. Occurrence of ultraviolet stabilizers in aquatic environments.

Composition	Sample location	Sample type	Concentrations	Key points/effects	Reference
**BUVSs**					
1BUVS<br>UV-360	3beaches(Gran Canaria island)	Seawater	41.12-544.9 ng/L	UV-360 was higher than other BUVSs.	(Garcia-Guerra et al. 2016)
Σ5BUVSs<br>(UV-P,UV-329,UV-350,UV-234,UV-328)	4 WWTPs and 4rivers (Shandongprovince, China)	Influent<br>Effluent<br>Water	0.66-37.1 ng/L<br>0.46-15.9 ng/L<br>ND-8.1ng/L	The different BUVSs concentrations and profiles among the different WWTP samples may indicate different usage of the UV stabilizers among the sampling regions.	(Liu et al. 2014)
Σ6BUVSs<br>(UV-9,UV-P,UV-326,UV-327,UV-328,UV-329)	Kaveri, Vellar, and Thamiraparani (Tamil Nadu, India)	Water	1.7-31.3 ng/L	UV-9 were found predominant in the study matrices during wet and dry season.	(Vimalkumar et al. 2018)
Σ4BTRs<br>(1H-BTR,Me-1HBTR,5,6-DIMe BTR,5-Cl-BTR)	5 STPs<br>Indian	Influent<br>Effluent	0.6-503 ng/L<br>3-42 ng/L	BTH levels were among thehighest ever reported in the literature, and the overall abundance of the target chemicals in wastewater was in thefollowing order: BTHs>> BTRs > BzPs > BPs	(Karthikraj and Kannan 2017)
Σ5BUVSs<br>(UV-P,UV-320,UV-326,UV-327,UV-328)	WWTP<br>Lisbon(Portugal)	Influent<br>Effluent	24-85 ng/L<br>ND-31 ng/L	Although Tinuvins appeared to be conveniently removed from the water phase in sewage treatment	(Carpinteiro et al.

	WWTP<br>Spain	Influent<br>Effluent	ND-65 ng/L<br>ND	plants, further studies are required to address their potential concentration in sludge.	2012)
Σ5BUVSs<br>(UV-P,UV-326,UV-327,UV-328,UV-329)	coastal region and offshore region of South China Sea	Seawater<br>coastal region<br>offshore region	 0.1-26.1 ng/L<br>ND-14.6 ng/L	Both coastal seawater and corals generally showed higher OUVA levels than offshore.	(Pei et al. 2023)
Σ4BUVSs<br>(1H-benzotriazole,1-hydroxy-benzotriazole,tolyltriazole, and xylyltriazole ,)	WWTP<br>Athens<br>(Greece)	Influent<br>Effluent	106-15841 ng/L<br>19-5737 ng/L	Occurrence of BTRs and BTHs was proved in the particulate matter of wastewater.	(Asimakopoulos et al. 2013)
Σ3UV filters<br>(BZT,MeBZT,DMBZT)	Adrià del Besòs<br>(NE of Spain)	Surfacewater<br>Groundwater	61.1-1340 ng/L<br>59.7-1980 ng/L	Triazoles (BZT and MeBZT) seemed to be the most persistent PCPs in the aquifer.	(Serra-Roig et al. 2016)
Σ9BUVSs<br>(UV-P,UV-531,UV-329,UV-320,UV-234,UV-326,UV-328,UV-327,UV-928)	South Korea	Seawater	0.172-1.75 ng/L	UV-326 can both bioaccumulate and biomagnify.	(Wang, Lee, and Oh 2022)
Σ9BUVSs<br>(UV-320，UV-350，UV-326，UV-329，UV-328，UV-327，UV-234，UV-9，UV-P)	Lake St. Louis (LSL), ÎlesdeBoucherville (IB),Îlet Vert (IV)<br>(St. Lawrence River,	LSL:Water<br>IB:Water<br>IV:Water	ND-13 ng/L<br>ND-92 ng/L<br>ND-12 ng/L	SPM showed greater sorption capacities of most target contaminants compared to those of the sediment.	(Dalpe Castilloux et al. 2022)

	Quebec, Canada)			The accumulation of UVAs and IAs in fish depends on their feeding behavior.	
Σ5BUVSs (UV-328,UV-329,UV-326,UV-234,UV-327)	9WWTPs (Canada）	Influent<br>Effluent	ND-34.4 ng/L<br>ND-2.61 ng/L	Wastewater effluent is a vector of SDPAs and BZT-UVs to the aquatic environment.<br>The results also highlight the high concentrations of SDPAs and BZT-UVs associated with the solid stream in WWTPs, which could affect the beneficial use of biosolids (e.g., compost or land applications).	(Lu, Smyth, et al. 2017)
Σ6BUVSs (UV-329,UV-328,UV-234,UV-326,UV-327,UV-350)	southern Ontario (Canada)	Upstream：Water<br>Downstrea：Water	＜0.05-84.3 ng/L<br>＜0.05-1.5 ng/L	SDPAs and BZT-UVs were frequently detected , suggesting a ubiquitous presence and bioaccumulation of these emerging contaminants.<br>Crayfish could be used as an indicator species for these compounds.	(Lu et al. 2016)
Σ6BUVSs (UV-9，UV326，UV-328，UV-P，UV-327，UV-329)	11WWTPs Tamil Nadu, India.	Influent<br>Effluent	65-567 ng/L<br>7.2-2732 ng/L	The wider usage of target ECs impregnated products in India and their elevated levels confirm their ubiquitous occurrence in wastewater.	(Vimalkumar et al. 2022)

				A personal care chemical (UV-328) may cause health implications in organisms of the effluent receiving systems.	
Σ4BUVSs (UV-324,UV-327,UV-328,UV-329)	3WWTPs (Norway)	Effluent (ng/L)	＜10 ng/L	It is possible that under certain low flow conditions selected organic UV filters may pose a risk to surface waters but under the present conditions the risk is low, but some UV filters will potentially accumulate through the trophic food chain.	(Langford , Reid, Fjeld, Øxnevad, et al. 2015)
Σ6BUVSs (UV-P,UV-326,UV-327,UV-328,UV-329,UV-360)	5WWTPs Gran Canaria (Canary Islands, Spain)	Influent<br>Effluent<br>Seawater	13.12-1933 ng/L<br>28.8-570.9 ng/L<br>67.01-2419 ng/L	The studied compounds exhibited different trends of occurrence in aqueous and solid samples due to their different coefficients of hydrophobicity.	(Montesdeoca-Esponda et al. 2019)
BTri	WWTPs、Lakes Canton of Zurich, Switzerland, Swiss midland region.	Influent<br>Effluent<br>Surface water<br>Groundwater	10-24 ug/L<br>4-12 ug/L<br>11-917 ng/L<br>16-77 ng/L	Concentration ratios between compounds indicated some elimination of BTri and DiOH-CBZ during subsurface passage or in groundwater, while CBZ and PMD appeared to be more stable and thus are promising wastewater markers for groundwater.	(Kahle et al. 2009)

Σ3BUVSs (MeBZT,BZT,UV-P)	Tunisia	Seawater	15.2-50.9 ng/L	MePB and MeBZT showed the highest sediment-water partition coefficients, tending to accumulate in sediments.	(Fenni et al. 2022)
Σ7BUVSs (UV-P,UV-329,UV-326,UV-328,UV-327,UV-571,UV-360)	3WWTPs Gran Canaria Island (Spain)	Seawater Wastewater	19.9-92.2 ng/L 19.8-83.3 ng/L		(Montesdeoca-Esponda et al. 2013)
Σ4BUVSs (BTri,5-TTri,5-ClBTri,XTri)	upstream and downstream of Xiangjiang River,China	Water	ND-21.7 ng/L	The monitoring of selected parabens, benzotriazoles, and antimicrobials in the Xiangjiang River showed that these compounds were more detected whereas benzophenones were less detected.The major three contaminants were MeP, PrP, and BTri.	(Lu et al. 2018)
Σ2BUVSs (UV-328,UV360)	3WWTPs Gran Canaria Island (Spain)	Sewage	17.3-99.2 ng/ml	The application of the proposed method has allowed detecting two compounds, UV 360 and UV 328 in the three analysed sewage samples, revealing higher concentrations of both compounds in the WWTP that only conducted	(Montesdeoca-Esponda et al. 2015)

				primary and secondary treatment.	
Σ5BUVSs (UV-P,Allyl-bzt,UV-326,UV-328,UV-327)	Laboratory	Wastewater	ND-57 ng/L	This fact confirms the contribution of sewage water to the introduction of UV stabilizers in the aquatic environment, where they are likely fixed to suspended particles and sediments.	(Carpinteiro et al. 2010)
Σ5BUVSs (UV-P,UV-329,UV-234,UV-326,,UV-328,)	WWTP (Jinan city, Shandong Province in China,)	Water	4.92-50.3 ng/L	UV-P, UV-326, UV-328 and UV-329 were considered as major BZT contaminants. Waste sludge could be a potential source of BZT-UVs reaching the environment.	(Song et al. 2014)
Σ7BUVSs (UVP,UV329,UV326,UV328,UV327, UV571 ,UV360)	7WWTPs,6beaches Gran Canaria Island (Spain).	Seawater<br>Wastewater	2.8-5.2 ng/L<br>4-13 ng/L		(Montesdeoca-Esponda, Sosa-Ferrera, and Juan Santana-Rodriguez 2012)

Σ9BUVSs (UV-P,UV-PS,UV-320,UV-350,UV-326,UV-329,UV-328,UV-327,UV-234)	WWTP Songhua River in Harbin Section, Northeastern China.	Wastewater	0-38.89 ng/L	The A/O treatment process provided better removal of BTs UV filters than the BAF treatment process. Seasonal changes have no significant influence on residual concentrations and removal efficiency.<br>The comparison with concentration and composition between upstream and downstream surface water indicated the influence of effluent discharge on the receiving water system.	(Zhao et al. 2017)
Σ11BUVSs (BTR,5-Cl-BTR,UV-P,UV-PS,UV-320,UV-350,UV-326,UV-329,UV-328,UV-327,UV-234)	Yangtze River (China)	Seawater	ND-10.73 ng/L	The main species were UV-P, UV-PS, UV-329, and UV-328.	(Zhao et al. 2023)
Σ4BUVSs (UV-320,UV-326,UV-329,UV-327)	Chaohu Lake.	Water	0.81-3.04 ng/L	High concentrations of BP were universally detected in the investigated water samples. A source assessment suggested the discharge from industries using UVAs as stabilizers to be an important source of their occurrence in aquatic environments, in addition to the	(Tang et al. 2018)

				direct source of domestic sewage resulting from the wide use of UVAs in cosmetics.	
5BUVSs (UV-P,UV-326,UV-327,UV-328,UV-329)	Pearl River basin (West River and North River) in Guangdong and Guangxi Province, south China	Surface water	0.65-59.8 ng/L	Significant seasonal differences of organic UV filters and BTs were found in surface water, but not in sediments.	(Hu et al. 2021)
6BUVSs (UV-P,UV-320,UV-326,UV-329,UV-328,UV-327)	3Rivers (India)	Water	ND-6.79 ng/L	UV-329 and UV-327 were the dominant BUVs in sediment and water.<br>BUVs posed low risk to planktons in water bodies.<br>Plastic debris and wastewater discharge were major sources of BUVs.	(Khare et al. 2023)
Σ2BUVSs (BZT,MeBZT)	20WWTPs Catalonia (Spain)	BZT:Influent<br>Effluent<br>MeBZT:Influent<br>Effluent	76.5-4380 ng/L<br>26.7-1513.8 ng/L<br>778.7-47142.9 ng/L<br>587.1-10541.2 ng/L	BZT and MeBZT were found in all the studied samples showing the widespread occurrence of these two contaminants.<br>MeBZT may represent a hazard for different aquatic organisms.	(Molins-Delgado, Silvia Diaz-Cruz, and Barcelo 2015)

Σ2BUVSs<br>(BZT,MeBZT)	6WWTPs、2Rivers.<br>Llobregat River basin and the Besòs River basin.<br>(Spain).	Influent<br>Effluent<br>River water	1495.2-9481.6ng/L<br>1084.1-16933.1ng/L<br>23.7-8529.8ng/L	BZT and MeBZT concentrations measured in some effluent streams do pose a risk for D. galeata and V. fischeri.	(Molins-Delgado et al. 2017)
Σ4BUVSs<br>(UV-324,UV-327,UV-328,UV-329)	3WWTPS<br>(Norway)	Effluent	5ng/L	it is possible that under certain low flow conditions selected organic UV filters may pose a risk to surface waters but under the present conditions the risk is low, but some UV filters will potentially accumulate through the trophic food chain.	(Langford, Reid, Fjeld, Oxnevad, et al. 2015)
6BUVSs<br>(BT,5-TTri,CBT,XTri,UV-326,UV-329)	WWTP<br>(South Australia)	Ground water<br>Effluent	97.5-280ng/L<br>77.2-2206ng/L	The highest concentration of BT reached up to 2.2 ug/L in effluent samples, indicating its persistence of BT in the environment.	(Liu et al. 2011)
Σ3BUVSs<br>(UV-329,UV-234,UV-327)	Bohai Sea (China), Qinglong Lake (Tangshan, China), and the North China University of Science and Technology (Tangshan, China)	Seawater<br>Lake water<br>Wastewater	2.9-43.7ng/ml<br>3.4-19.3ng/ml<br>ND		(Zhang et al. 2023)

Sediment					
Σ6BUVSs (UV-9,UV-P,UV-326,UV-327,UV-328,UV-329)	Kaveri, Vellar, and Thamiraparani (Tamil Nadu, India)	Sediment	0.2-7.3ng/g dw	UV-9 were found predominant in the study matrices during wet and dry season.	(Vimalkumar et al. 2018)
Σ4BUVSs (UV-320,UV-326,-UV-327,UV-328)	Ariake Sea, Japan	Sediment	0.3-320ng/g dw	UV-327 was the most frequently detected compound in organisms analyzed.	(Nakata, Murata, and Filatreau 2009)
Σ3BUVSs (BZT,TBHPBT,MeBZT)	Palmital River, Atuba River, Belem River, Iguaçu River (Upper Iguaçu watershed, Curitiba (Brazil))	Sediment<br>Palmital River<br>Atuba River<br>Belem River<br>Iguaçu River	 6.7-630ng/g dw<br>5.7-322.2ng/g dw<br>9.3-313.8ng/g dw<br>4.6-160.8ng/g dw	Both hydrophobic and polar compounds are accumulated in the sediments.<br>Urban wastewater is the main source of the contamination observed in the sediments.	(Mizukawa et al. 2017)
Σ9BUVSs (UV-P,UV-234,UV-320,UV-326,UV-327,UV-328,UV-329,UV-350,UV-360)	GermanBight, Skagerrak and Kattegat,German Baltic Sea (European North and Baltic Seas)	Sediment<br>GermanBight:<br>Skagerrak and Kattegat:<br>German Baltic Sea	 ND-0.13ng/g dw<br>ND-0.1ng/g dw<br>ND-0.63ng/g dw	Bisoctrizole (UV-360) was the predominant benzotriazole UV stabilizer.	(Apel, Joerss, and Ebinghaus 2018)

Σ9BUVSs (UV-P,UV-531,UV-329,UV-320,UV-234,UV-326,UV-328,UV-327,UV-928)	South Korea	Sediment	ND-0.422ng/g dw	UV-326 can both bioaccumulate and biomagnify.	(Wang, Lee, and Oh 2022)
Σ9BUVSs (UV-320，UV-350，UV-326，UV-329，UV-328，UV-327，UV-234，UV-9，UV-P)	Lake St. Louis (LSL), ÎlesdeBoucherville (IB),Îlet Vert (IV) (St. Lawrence River, Quebec, Canada)	LSL:Sediment<br>IB:Sediment<br>IV:Sediment	ND-20ng/g dw<br>ND-16ng/g dw<br>ND-43ng/g dw	SPM showed greater sorption capacities of most target contaminants compared to those of the sediment.<br>The accumulation of UVAs and IAs in fish depends on their feeding behavior.	(Dalpe Castilloux et al. 2022)
Σ2BUVSs (BZT,MeBZT)	6WWTPs、2Rivers. Llobregat River basin and the Besòs River basin.<br>(Spain).	Sediment	ND-230.9ng/g dw	BZT and MeBZT concentrations measured in some effluent streams do pose a risk for D. galeata and V. fischeri.	(Molins-Delgado et al. 2017)
Σ6BUVSs (UV-329,UV-328,UV-234,UV-326,UV-327,UV-350)	southern Ontario (Canada)	Upstream：Sediment<br>Downstrea：Sediment	＜0.01-0.7ng/g dw<br>＜0.08-3ng/g dw	SDPAs and BZT-UVs were frequently detected , suggesting a ubiquitous presence and bioaccumulation of these emerging contaminants.<br>Crayfish could be used as an	(Lu et al. 2016)

				indicator species for these compounds.	
Σ4BUVSs (UV-324,UV-327,UV-328,UV-329)	3WWTPs (Norway)	Sediment	＜65ng/g dw	It is possible that under certain low flow conditions selected organic UV filters may pose a risk to surface waters but under the present conditions the risk is low, but some UV filters will potentially accumulate through the trophic food chain.	(Langford , Reid, Fjeld, Øxnevad, et al. 2015)
Σ9BUVSs (UV-326,UV-320,UV-329, UV-350, UV-328,UV-327,UV-928, UV-234，UV-360)	5Rivers (German)	Sediment	0.23-21ng/g dw	Five of the nine examined BUVSs were also detected in bream liver and confirmed their high bioaccumulation potential. This supports the so far only predicted high persistence of BUVSs in aquatic systems. High persistence of BUVSs in aquatic systems.	(Wick et al. 2016)
Σ6BUVSs (UV-P,UV-326,UV-327,UV-328,UV-329,UV-360)	5WWTPs Gran Canaria (Canary Islands, Spain)	Sediments	4.42-2162ng/kg dw	The studied compounds exhibited different trends of occurrence in aqueous and solid samples due to their different coefficients of hydrophobicity.	(Montesdeoca-Esponda et al.

					2019)
Σ7BUVSs (UV234;UV320;UV326;UV327;UV328;UV329)	Bohai and Yellow Seas(China)	Sediment	Bohai：0.022-0.17ng/g dw<br>Yellow Sea:0.01-0.83ng/g dw	UV-329 and UV-326 may have an identical input pathway and similar environmental behavior.	(Apel, Tang, and Ebinghaus 2018)
MeBZT	Tunisia	Sediment	8.7-24.9ng/g dw	MePB and MeBZT showed the highest sediment-water partition coefficients, tending to accumulate in sediments.	(Fenni et al. 2022)
Σ4BUVSs (UV-329,UV-326,UV-327,UV-320)	5Rivers Tianjin, China	Sediment	1.09-1.35mg/g dw	Source assessment further suggested that the industrial activities seemly contributed more to the sedimentOUVAs than the use of cosmetics and PCPs in the area.	(Li, Xing, et al. 2022)
Σ11BUVSs (BTR,5-Cl-BTR,UV-P,UV-PS,UV-320,UV-350,UV-326,UV-329,UV-328,UV-327,UV-234)	Yangtze River (China)	Sediment	ND-11.31ng/g	The main species were UV-P, UV-PS, UV-329, and UV-328.	(Zhao et al. 2023)
Σ4BUVSs (UV-320,UV-326,UV-329,UV-327)	Chaohu Lake.	Sediment	＜0.15-0.64ng/g	High concentrations of BP were universally detected in the investigated water samples. A source assessment suggested the	(Tang et al. 2018)

				discharge from industries using UVAs as stabilizers to be an important source of their occurrence in aquatic environments, in addition to the direct source of domestic sewage resulting from the wide use of UVAs in cosmetics.	
Σ5BUVSs (UV-P,UV-326,UV-327,UV-328,UV-329)	Pearl River basin (West River and North River) in Guangdong and Guangxi Province, south China	Sediment	3.82-18.8ng/g	Significant seasonal differences of organic UV filters and BTs were found in surface water, but not in sediments.	(Hu et al. 2021)
Σ6BUVSs (UV-P,UV-320,UV-326,UV-329,UV-328,UV-327)	3Rivers (India)	Sediment	ND-16.1ng/g	UV-329 and UV-327 were the dominant BUVs in sediment and water.<br>BUVs posed low risk to planktons in water bodies.<br>Plastic debris and wastewater discharge were major sources of BUVs.	(Khare et al. 2023)
Σ4BUVSs (UV-324,UV-327,UV-328,UV-329)	3WWTPS (Norway)	Sediment	4.8-12.5ng/g dw	it is possible that under certain low flow conditions selected organic UV filters may pose a risk to surface waters but under the	(Langford , Reid, Fjeld,

				present conditions the risk is low, but some UV filters will potentially accumulate through the trophic food chain.	Oxnevad, et al. 2015)
Sludge					
Σ4BTRs (1H-BTR,Me-1HBTR,5,6-DIMe BTR,5-Cl-BTR)	5 STPs Indian	Sludge	0.8-58.4ng/g dw	BTH levels were among thehighest ever reported in the literature, and the overall abundance of the target chemicals in wastewater was in thefollowing order: BTHs>> BTRs > BzPs > BPs	(Karthikraj and Kannan 2017)
Σ9BUVSs (UV-P,UV-PS,UV-329,UV-350,UV-320,UV-234,UV-326,UV-328,UV-327)	WWTPs (33 cities,China)	Sludge	ND-24700ng/g dw	The most dominant analogue was UV-234and accounted on average for 27.2% of total BZTs. Significant correlations were found among the BZT concentrations and also with a WWTP characteristic (daily treatment volume). The commercial BZT chemicals and their plausible transformation products might be persistent in the environment.	(Ruan et al. 2012)

Σ4BUVSs (1H-benzotriazole,1-hydroxy-benzotriazole,tolyltriazole, and xylyltriazole ,)	WWTP Athens (Greece)	Sludge	25-116ng/g	Occurrence of BTRs and BTHs was proved in the particulate matter of wastewater.	(Asimakopoulos et al. 2013)
Σ4BUVSs (UV-324,UV-327,UV-328,UV-329)	3WWTPs (Norway)	Sludge	1172-3303ng/g dw	It is possible that under certain low flow conditions selected organic UV filters may pose a risk to surface waters but under the present conditions the risk is low, but some UV filters will potentially accumulate through the trophic food chain.	(Langford, Reid, Fjeld, Øxnevad, et al. 2015)
Biota					
Σ6BUVSs (UV-9,UV-P,UV-326,UV-327,UV-328,UV-329)	Kaveri, Vellar, and Thamiraparani (Tamil Nadu, India)	Fish	0.3-79.4ng/g ww	UV-9 were found predominant in the study matrices during wet and dry season.	(Vimalkumar et al. 2018)
Σ4BUVSs (UV-320,UV-326,-UV-327,UV-328)	Ariake Sea, Japan	Marine Organisms	＜0.05-55ng/g ww	UV-327 was the most frequently detected compound in organisms analyzed.	(Nakata, Murata, and Filatreau 2009)

Σ7BUVSs (UV-P,UV-234,UV-320,UV-326,UV-327 ,UV-328 ,UV-329)	BohaiSea (China)	mollusks	ND-110ng/g dw	UV-326, UV-327, UV-Pwere thepredominant analogues, andUV-328 wasthemost frequently detected BZT-UV.	(Li, Su, et al. 2022)
Σ3BUVSs (UV-320,UV-327,UV-328)	Ariake Sea regions, western Japan	Finless porpoises	<0.05-64ng/g ww	BUVSs appear to be persistent and bioaccumulative in the aquatic food chain.	(Nakata et al. 2010)
Σ9BUVSs (UV-P,UV-531,UV-329,UV-320,UV-234,UV-326,UV-328,UV-327,UV-928)	South Korea	Fish<br>Benthic invertebrate	30.9-281ng/g Lw<br>35.3-228ng/g Lw	UV-326 can both bioaccumulate and biomagnify.	(Wang, Lee, and Oh 2022)
Σ5BUVSs (UV-328,UV-329,UV-326,UV-234,UV-327)	9WWTPs (Canada）	Biosolids	ND-239ng/g dw	Wastewater effluent is a vector of SDPAs and BZT-UVs to the aquatic environment.<br>The results also highlight the high concentrations of SDPAs and BZT-UVs associated with the solid stream in WWTPs, which could affect the beneficial use of biosolids (e.g., compost or land applications).	(Lu, Smyth, et al. 2017)

Σ6BUVSs (BT,5-TTri,CBT,XTri,UV-326,UV-329)	WWTP (South Australia)	Biosolid	＜2.9-21.4ng/g	The highest concentration of BT reached up to 2.2 ug/L in effluent samples, indicating its persistence of BT in the environment.	(Liu et al. 2011)
Σ6BUVSs (UV-329,UV-328,UV-234,UV-326,UV-327,UV-350)	southern Ontario (Canada)	Upstream：<br>Biota:Crayfish<br>Chub<br>Shiner<br>Downstrea：<br>Biota:Crayfish<br>Chub<br>Shiner	 ＜0.2-1076ng/g Lw<br>＜0.07-6.86ng/g Lw<br>＜0.07-2ng/g Lw<br><br>＜0.2-1306ng/g Lw<br>＜0.004-8.1ng/g Lw<br>＜0.07-3.86ng/g Lw	SDPAs and BZT-UVs were frequently detected , suggesting a ubiquitous presence and bioaccumulation of these emerging contaminants.<br>Crayfish could be used as an indicator species for these compounds.	(Lu et al. 2016)
Σ4BUVSs (UV-324,UV-327,UV-328,UV-329)	3WWTPs (Norway)	Biota:Cod (liver)<br>Prawn(whole)<br>Crab (whole)<br>Burbot (fillet)<br>Whitefish(fillet)<br>Perch (fillet)	＜10-＜50ng/g<br>＜10–51.8ng/g<br>＜25ng/g<br>＜50ng/g<br>＜50ng/g<br>＜25ng/g	It is possible that under certain low flow conditions selected organic UV filters may pose a risk to surface waters but under the present conditions the risk is low, but some UV filters will potentially accumulate through the trophic food chain.	(Langford , Reid, Fjeld, Øxnevad, et al. 2015)
Σ4BUVSs (UV-320,UV-326.UV-327,UV-328)	Cambodia<br>China<br>Hong Kong<br>India<br>Indonesia	Mussels	＜0.05-120<br>＜0.05-96<br>＜0.05-200<br>＜0.05<br>＜0.05-120	Significant correlations were found between the concentrations of HHCB and AHTN, and also between the concentrations of UV-327 and UV-328, which suggest	(Nakata et al. 2012)

	Japan		33-450	similar sources and compositions of these compounds in commercial and industrial products.	
	Korea		＜0.05-220		
	Malaysia		＜0.05-42		
	Philippines		＜0.05-170		
	USA		＜0.05-130		
	Vietnam		＜0.05		
Σ4BUVSs (UV-326,UV-328,UV-329,UV-360)	Gran Canaria Island (Spain)	fish samples：<br>Boops boops,<br>Sphyraena viridensis，<br>Sphoeroides Marmoratus，	Muscle：5.2-34.9ng/g dw<br>Viscera：2.3-45.6ng/g dw<br>Muscle：6.9ng/g dw<br>Viscera ： 2.16-34.5ng/g dw<br>Muscle：4.16-16.8ng/g dw<br>Viscera ： 1.34-10.7ng/g dw	These results demonstrate the widespread dissemination of BUVSs in the marine ecosystem.	(Montesdeoca-Esponda et al. 2020)
Σ8BUVSs (UV-P,UV-9,UV-234,UV-320 ,UV-326 ,UV-327,UV-328 ,UV-329)	Manila Bay, the Philippines.	Fishes	0.02-211ng/g Lw	Among BUVSs, UV-P showed significant positive relationship between concentration and fish length and fish weight .<br>Possibile sink of UV-P is bottom sediment in the bay, and ultimately	(Kim, Isobe, et al. 2011)

				accumulate through benthic food web rather than pelagic food web.	
Σ8BUVSs (UV-P,UV-9,UV-234,UV-320,UV-326,UV-327,UV-328,UV-329)	Manila Bay, the Philippines	Fish	<0.0002-179ng/g LW	The ubiquitous contamination by these emerging pollutants in Manila Bay.	(Kim, Ramaswamy, et al. 2011)
Σ4BUVSs (UV-320,UV-326,UV-329,UV-327)	Lake Chaohu,China.	Fish	＜0.17-6.23ng/g dw	High liver accumulation capacity of UVAs was observed in fish.	(Tang et al. 2019)
Σ4BUVSs (BT,5-TT,CBT,XT)	Dongjing River,China	Fish muscle<br>liver	＜0.21-3.88ng/g ww<br>ND-33.7ng/g ww	Most of the target compounds showed high detection frequencies, indicating bioaccumulation of these chemicals by wild fish. The concentrations for most analytes in liver tissue were higher than those in muscle tissue.	(Yao et al. 2016)
Σ2BUVSs (UV-234,UV-328)	Ontario, Canada	Fish	0.219-4.9ng/g ww	The concentrationof target compounds generally decreased in the following order: liver > carcass homogenate ⩾ bile > plasma. This indicates that liver is a major tissue for accumulation of these contaminants in fish.	(Lu, De Silva, et al. 2017)

Others					
Σ9BUVSs (UV-P,UV-PS,UV-9,UV-320,UV-326,UV-327,UV-328,UV-329,UV-350)	Tokyo (Japan)	Plastic products<br>Beveragebottlecaps<br>food packages<br>shopping bags	 0.04-160ng/g<br>0.1-27.7ng/g<br>0.13-209ng/g	UV-P,UV-PS,UV-090,UV-329 showed ERα and/ or ERagonistic activities. UV-320,UV-350 acted as both ERα/β antagonists, and UV-328/-329 acted as ERβ antagonists.UV-P,UV-9 showed AR antagonistic activity. plastic additives, BUVSs, are present in our daily environment and several of them have endocrine-disrupting effects via ERs and AR.	(Sakuragi et al. 2021)
Σ5BUVSs (UV-234,UV-328,UV-P,1-H-benzotriazole,5-Methyl-benzotriazole)	Philippines, Indonesia, Switzerland, India, Georgia, China, Cambodia, Egypt, Bangladesh, Portugal, Turkey,	Clothing	1.44-3120ng/g	Clothing textiles can be a possible route for human exposure to harmful chemicals by skin contact, as well as being a potential source of environmental pollutants via laundering and release to household wastewater.	(Avagyan et al. 2015)

	Bulgaria, Latvia, Lithuania				
Σ9BUVSs (UV-320，UV-350，UV-326，UV-329，UV-328，UV-327，UV-234，UV-9，UV-P)	Lake St. Louis (LSL), ÎlesdeBoucherville (IB),Îlet Vert (IV) (St. Lawrence River, Quebec, Canada)	LSL:SPM<br>IB:SPM<br>IV:SPM	ND-499ng/g dw<br>ND-611ng/g dw<br>ND-235ng/g dw	SPM showed greater sorption capacities of most target contaminants compared to those of the sediment.<br>The accumulation of UVAs and IAs in fish depends on their feeding behavior.	(Dalpe Castilloux et al. 2022)
Σ2BUVSs (BZT,MeBZT)	6WWTPs、2Rivers. Llobregat River basin and the Besòs River basin. (Spain).	Suspended solid	416.8-21832.1ng/g dw	BZT and MeBZT concentrations measured in some effluent streams do pose a risk for D. galeata and V. fischeri.	(Molins-Delgado et al. 2017)
Σ4BUVSs (UV-324,UV-327,UV-328,UV-329)	3WWTPs (Norway)	Landfill leachate	＜5-19ng/L	It is possible that under certain low flow conditions selected organic UV filters may pose a risk to surface waters but under the present conditions the risk is low, but some UV filters will potentially accumulate through the trophic food chain.	(Langford , Reid, Fjeld, Øxnevad, et al. 2015)

Σ4BUVSs (UV-324,UV-327,UV-328,UV-329)	3WWTPS (Norway)	Landfill leachate	＜5-19ng/L	it is possible that under certain low flow conditions selected organic UV filters may pose a risk to surface waters but under the present conditions the risk is low, but some UV filters will potentially accumulate through the trophic food chain.	(Langford , Reid, Fjeld, Oxnevad, et al. 2015)
Σ4BUVSs (UV-320,UV-326,UV-327,UV-328)	South Korea	Marine debris	0.3-81700ng/g	The detection frequency and concentration levels of additive chemicals (except for Irganox 1076 and UV 320) in new plastics were relatively high in new plastics compared to marine debris, implying their potential leaching or degradation during use or after disposal.	(Rani et al. 2017)
Σ10BUVSs (UV-P,UV-PS,UV-329,UV-9,UV-320,UV-350,UV-326,UV-327,UV-328,UV-234)	Otsuchi town, Iwate Prefecture, Japan	propylene face masks	1.4-848ng/g	The face mask found in the feces of a green sea turtle.	(Fukuoka et al. 2022)

Apel, Christina, Hanna Joerss, and Ralf Ebinghaus. 2018. 'Environmental occurrence and hazard of organic UV stabilizers and UV filters in the sediment of European North and Baltic Seas', *Chemosphere*, 212: 254-61.

Apel, Christina, Jianhui Tang, and Ralf Ebinghaus. 2018. 'Environmental occurrence and distribution of organic UV stabilizers and UV filters in the sediment of Chinese Bohai and Yellow Seas', *Environmental Pollution*, 235: 85-94.

Asimakopoulos, Alexandros G., Akinranti Ajibola, Kurunthachalam Kannan, and Nikolaos S. Thomaidis. 2013. 'Occurrence and removal efficiencies of benzotriazoles and benzothiazoles in a wastewater treatment plant in Greece', *Science of the Total Environment*, 452: 163-71.

Avagyan, Rozanna, Giovanna Luongo, Gunnar Thorsen, and Conny Ostman. 2015. 'Benzothiazole, benzotriazole, and their derivates in clothing textiles-a potential source of environmental pollutants and human exposure', *Environmental Science and Pollution Research*, 22: 5842-49.

Carpinteiro, Inma, Maria Ramil, Isaac Rodriguez, and Jose Manuel F. Nogueira. 2012. 'Combining stir-bar sorptive extraction and large volume injection-gas chromatography-mass spectrometry for the determination of benzotriazole UV stabilizers in wastewater matrices', *Journal of Separation Science*, 35: 459-67.

Carpinteiro, Inmaculada, Brais Abuin, Isaac Rodriguez, Rafael Cela, and Maria Ramil. 2010. 'Headspace solid-phase microextraction followed by gas chromatography tandem mass spectrometry for the sensitive determination of benzotriazole UV stabilizers in water samples', *Analytical and Bioanalytical Chemistry*, 397: 829-39.

Dalpe Castilloux, Abigaelle, Magali Houde, Andree Gendron, Amila De Silva, Youssouf Djibril Soubaneh, and Zhe Lu. 2022. 'Distribution and Fate of Ultraviolet Absorbents and IndustrialAntioxidants in the St. Lawrence River, Quebec, Canada', *Environmental Science & Technology*, 56: 5009-19.

Fenni, Ferdaws, Adria Sunyer-Caldu, Hedi Ben Mansour, and M. Silvia Diaz-Cruz. 2022. 'Contaminants of emerging concern in marine areas: First evidence of UV filters and paraben preservatives in seawater and sediment on the eastern coast of Tunisia', *Environmental Pollution*, 309.

Fukuoka, T., F. Sakane, C. Kinoshita, K. Sato, K. Mizukawa, and H. Takada. 2022. 'Covid-19-derived plastic debris contaminating marine ecosystem: Alert from a sea turtle', *Marine Pollution Bulletin*, 175.

Garcia-Guerra, Romualdo B., Sarah Montesdeoca-Esponda, Zoraida Sosa-Ferrera, Abuzar Kabir, Kenneth G. Furton, and Jose Juan Santana-Rodriguez. 2016. 'Rapid monitoring of residual UV-stabilizers in seawater samples from beaches using fabric phase sorptive extraction and UHPLC-MS/MS', *Chemosphere*, 164: 201-07.

Hu, Li-Xin, Yu-Xiao Cheng, Dan Wu, Lei Fan, Jia-Hui Zhao, Qian Xiong, Quan-Le Chen, You-Sheng Liu, and Guang-Guo Ying. 2021. 'Continuous input of organic ultraviolet filters and benzothiazoles threatens the surface water and sediment of two major rivers in the Pearl River Basin', *Science of the Total Environment*, 798.

Kahle, M., I. J. Buerge, M. D. Muller, and T. Poiger. 2009. 'HYDROPHILIC ANTHROPOGENIC MARKERS FOR QUANTIFICATION OF WASTEWATER CONTAMINATION IN GROUND- AND SURFACE WATERS', *Environmental Toxicology and Chemistry*, 28: 2528-36.

Karthikraj, Rajendiran, and Kurunthachalam Kannan. 2017. 'Mass loading and removal of benzotriazoles, benzothiazoles, benzophenones, and bisphenols in Indian sewage treatment plants', *Chemosphere*, 181: 216-23.

Khare, Ankur, Pradip Jadhao, Shatabdi Kawre, Gajanan Kanade, Mahendra Patil, Atul Narayan Vaidya, and Asirvatham Ramesh Kumar. 2023. 'Occurrence, spatio-temporal variation and ecological

risk assessment of benzotriazole ultraviolet stabilizers (BUVs) in water and sediment of rivers in central India', *The Science of the total environment*: 163381-81.

Kim, Joon-Woo, Tomohiko Isobe, Babu Rajendran Ramaswamy, Kwang-Hyeon Chang, Atsuko Amano, Todd M. Miller, Fernando P. Siringan, and Shinsuke Tanabe. 2011. 'Contamination and bioaccumulation of benzotriazole ultraviolet stabilizers in fish from Manila Bay, the Philippines using an ultra-fast liquid chromatography-tandem mass spectrometry', *Chemosphere*, 85: 751-58.

Kim, Joon-Woo, Babu Rajendran Ramaswamy, Kwang-Hyeon Chang, Tomohiko Isobe, and Shinsuke Tanabe. 2011. 'Multiresidue analytical method for the determination of antimicrobials, preservatives, benzotriazole UV stabilizers, flame retardants and plasticizers in fish using ultra high performance liquid chromatography coupled with tandem mass spectrometry', *Journal of Chromatography A*, 1218: 3511-20.

Langford, Katherine H., Malcolm J. Reid, Eirik Fjeld, Sigurd Oxnevad, and Kevin V. Thomas. 2015. 'Environmental occurrence and risk of organic UV filters and stabilizers in multiple matrices in Norway', *Environment International*, 80: 1-7.

Langford, Katherine H., Malcolm J. Reid, Eirik Fjeld, Sigurd Øxnevad, and Kevin V. Thomas. 2015. 'Environmental occurrence and risk of organic UV filters and stabilizers in multiple matrices in Norway', *Environment International*, 80: 1-7.

Li, Pengyang, Wenyuan Su, Wenqing Liang, Bao Zhu, Tingyu Li, Ting Ruan, and Guibin Jiang. 2022. 'Occurrence and Temporal Trends of Benzotriazole UV Stabilizers in Mollusks (2010-2018) from the Chinese Bohai Sea Revealed by Target, Suspect, and Nontarget Screening Analysis', *Environmental Science & Technology*, 56: 16759-67.

Li, Yonghong, Xiangyang Xing, Di An, Jiazheng Sun, and Zhenwu Tang. 2022. 'Occurrence and distribution of organic ultraviolet absorbents in sediments from small urban rivers, Tianjin, China: Implications for risk management', *Ecotoxicology and Environmental Safety*, 230.

Liu, Runzeng, Ting Ruan, Thanh Wang, Shanjun Song, Feng Guo, and Guibin Jiang. 2014. 'Determination of nine benzotriazole UV stabilizers in environmental water samples by automated on-line solid phase extraction coupled with high-performance liquid chromatography–tandem mass spectrometry', *Talanta*, 120: 158-66.

Liu, You-Sheng, Guang-Guo Ying, Ali Shareef, and Rai S. Kookana. 2011. 'Simultaneous determination of benzotriazoles and ultraviolet filters in ground water, effluent and biosolid samples using gas chromatography-tandem mass spectrometry', *Journal of Chromatography A*, 1218: 5328-35.

Lu, Jing, Haipu Li, Zhoufei Luo, Huiju Lin, and Zhaoguang Yang. 2018. 'Occurrence, distribution, and environmental risk of four categories of personal care products in the Xiangjiang River, China', *Environmental Science and Pollution Research*, 25: 27524-34.

Lu, Zhe, Amila O. De Silva, Thomas E. Peart, Cyril J. Cook, and Gerald R. Tetreault. 2017. 'Tissue Distribution of Substituted Diphenylamine Antioxidants and Benzotriazole Ultraviolet Stabilizers in White Sucker (Catostomus commersonii) from an Urban Creek in Canada', *Environmental Science & Technology Letters*, 4: 433-38.

Lu, Zhe, Amila O. De Silva, Thomas E. Peart, Cyril J. Cook, Gerald R. Tetreault, Mark R. Servos, and Derek C. G. Muir. 2016. 'Distribution, Partitioning and Bioaccumulation of Substituted Diphenylamine Antioxidants and Benzotriazole UV Stabilizers in an Urban Creek in Canada', *Environmental Science & Technology*, 50: 9089-97.

Lu, Zhe, Shirley Anne Smyth, Thomas E. Peart, and Amila O. De Silva. 2017. 'Occurrence and fate of substituted diphenylamine antioxidants and benzotriazole UV stabilizers in various Canadian wastewater treatment processes', *Water Research*, 124: 158-66.

Mizukawa, Alinne, Daniel Molins-Delgado, Julio Cesar Rodrigues de Azevedo, Cristovao Vicente Scapulatempo Fernandes, Silvia Diaz-Cruz, and Damia Barcelo. 2017. 'Sediments as a sink for UV filters and benzotriazoles: the case study of Upper Iguacu watershed, Curitiba (Brazil)', *Environmental Science and Pollution Research*, 24: 18284-94.

Molins-Delgado, Daniel, M. Silvia Diaz-Cruz, and Damia Barcelo. 2015. 'Removal of polar UV stabilizers in biological wastewater treatments and ecotoxicological implications', *Chemosphere*, 119: S51-S57.

Molins-Delgado, Daniel, João Távora, M. Silvia Díaz-Cruz, and Damià Barceló. 2017. 'UV filters and benzotriazoles in urban aquatic ecosystems: The footprint of daily use products', *Science of the Total Environment*, 601-602: 975-86.

Montesdeoca-Esponda, Sarah, Cristina Álvarez-Raya, María Esther Torres-Padrón, Zoraida Sosa-Ferrera, and José Juan Santana-Rodríguez. 2019. 'Monitoring and environmental risk assessment of benzotriazole UV stabilizers in the sewage and coastal environment of Gran Canaria (Canary Islands, Spain)', *Journal of Environmental Management*, 233: 567-75.

Montesdeoca-Esponda, Sarah, Adrian del Toro-Moreno, Zoraida Sosa-Ferrera, and Jose Juan Santana-Rodriguez. 2013. 'Development of a sensitive determination method for benzotriazole UV stabilizers in enviromental water samples with stir bar sorption extraction and liquid desorption prior to ultra-high performance liquid chromatography with tandem mass spectrometry', *Journal of Separation Science*, 36: 2168-75.

Montesdeoca-Esponda, Sarah, Maria Esther Torres-Padron, Martin Novak, Lucie Krchova, Zoraida Sosa-Ferrera, and Jose Juan Santana-Rodriguez. 2020. 'Occurrence of benzotriazole UV stabilizers in coastal fishes', *Journal of Environmental Management*, 269.

Montesdeoca-Esponda, Sarah, Zoraida Sosa-Ferrera, and Jose Juan Santana-Rodriguez. 2012. 'On-line solid-phase extraction coupled to ultra-performance liquid chromatography with tandem mass spectrometry detection for the determination of benzotriazole UV stabilizers in coastal marine and wastewater samples', *Analytical and Bioanalytical Chemistry*, 403: 867-76.

Montesdeoca-Esponda, Sarah, Zoraida Sosa-Ferrera, Abuzar Kabir, Kenneth G. Furton, and Jose Juan Santana-Rodriguez. 2015. 'Fabric phase sorptive extraction followed by UHPLC-MS/MS for the analysis of benzotriazole UV stabilizers in sewage samples', *Analytical and Bioanalytical Chemistry*, 407: 8137-50.

Nakata, Haruhiko, Sayaka Murata, and Julien Filatreau. 2009. 'Occurrence and Concentrations of Benzotriazole UV Stabilizers in Marine Organisms and Sediments from the Ariake Sea, Japan', *Environmental Science & Technology*, 43: 6920-26.

Nakata, Haruhiko, Ryu-ichi Shinohara, Sayaka Murata, and Mafumi Watanabe. 2010. 'Detection of benzotriazole UV stabilizers in the blubber of marine mammals by gas chromatography-high resolution mass spectrometry (GC-HRMS)', *Journal of Environmental Monitoring*, 12: 2088-92.

Nakata, Haruhiko, Ryu-Ichi Shinohara, Yusuke Nakazawa, Tomohiko Isobe, Agus Sudaryanto, Annamalai Subramanian, Shinsuke Tanabe, Mohamad Pauzi Zakaria, Gene J. Zheng, Paul K. S. Lam, Eun Young Kim, Byung-Yoon Min, Sung-Ug We, Viet Pham Hung, Touch Seang Tana, Maricar Prudente, Donnell Frank, Gunnar Lauenstein, and Kurunthachalam Kannan. 2012. 'Asia-Pacific

mussel watch for emerging pollutants: Distribution of synthetic musks and benzotriazole UV stabilizers in Asian and US coastal waters', *Marine Pollution Bulletin*, 64: 2211-18.

Pei, Jiying, Junjie Hu, Ruijie Zhang, Nai Liu, Wenfeng Yu, Annan Yan, Minwei Han, Huanxin Liu, Xueyong Huang, and Kefu Yu. 2023. 'Occurrence, bioaccumulation and ecological risk of organic ultraviolet absorbers in multiple coastal and offshore coral communities of the South China Sea', *Science of the Total Environment*, 868.

Rani, Manviri, Won Joon Shim, Gi Myung Han, Mi Jang, Young Kyoung Song, and Sang Hee Hong. 2017. 'Benzotriazole-type ultraviolet stabilizers and antioxidants in plastic marine debris and their new products', *Science of the Total Environment*, 579: 745-54.

Ruan, Ting, Runzeng Liu, Qiang Fu, Wang Thanh, Yawei Wang, Shanjun Song, Pu Wang, Man Teng, and Guibin Jiang. 2012. 'Concentrations and Composition Profiles of Benzotriazole UV Stabilizers in Municipal Sewage Sludge in China', *Environmental Science & Technology*, 46: 2071-79.

Sakuragi, Yuuta, Hideshige Takada, Hiroya Sato, Atsuhito Kubota, Masaru Terasaki, Shinji Takeuchi, Atsuko Ikeda-Araki, Yoko Watanabe, Shigeyuki Kitamura, and Hiroyuki Kojima. 2021. 'An analytical survey of benzotriazole UV stabilizers in plastic products and their endocrine-disrupting potential via human estrogen and androgen receptors', *Science of the Total Environment*, 800.

Serra-Roig, Maria Pau, Anna Jurado, M. Silvia Díaz-Cruz, Enric Vázquez-Suñé, Estanislao Pujades, and Damià Barceló. 2016. 'Occurrence, fate and risk assessment of personal care products in river–groundwater interface', *Science of the Total Environment*, 568: 829-37.

Song, Shanjun, Ting Ruan, Thanh Wang, Runzeng Liu, and Guibin Jiang. 2014. 'Occurrence and removal of benzotriazole ultraviolet stabilizers in a wastewater treatment plant in China', *Environmental Science-Processes & Impacts*, 16: 1076-82.

Tang, Zhenwu, Xue Han, Guanghui Li, Shulei Tian, Yufei Yang, Fuyong Zhong, Yu Han, and Jun Yang. 2018. 'Occurrence, distribution and ecological risk of ultraviolet absorbents in water and sediment from Lake Chaohu and its inflowing rivers, China', *Ecotoxicology and Environmental Safety*, 164: 540-47.

Tang, Zhenwu, Fuyong Zhong, Jiali Cheng, Zhiqiang Nie, Xue Han, Yu Han, and Yufei Yang. 2019. 'Concentrations and tissue-specific distributions of organic ultraviolet absorbents in wild fish from a large subtropical lake in China', *Science of the Total Environment*, 647: 1305-13.

Vimalkumar, Krishnamoorthi, Elaiyaraja Arun, Selvaraj Krishna-Kumar, Rama Krishnan Poopal, Nishikant Patil Nikhil, Annamalai Subramanian, and Ramaswamy Babu-Rajendran. 2018. 'Occurrence of triclocarban and benzotriazole ultraviolet stabilizers in water, sediment, and fish from Indian rivers', *Science of the Total Environment*, 625: 1351-60.

Vimalkumar, Krishnamoorthi, Murugasamy Mayilsamy, Elayaraja Arun, Balasubramanian Gobinath, Saravanan Prasanth, Patil Nishikant Nikhil, Selvaraj Krishna-Kumar, Sampath Srimurali, Martin Mkandawire, and Ramaswamy Babu-Rajendran. 2022. 'Screening of antimicrobials, fragrances, UV stabilizers, plasticizers and preservatives in sewage treatment plants (STPs) and their risk assessment in India', *Chemosphere*, 308.

Wang, Wenting, In-Seok Lee, and Jeong-Eun Oh. 2022. 'Specific-accumulation and trophic transfer of UV filters and stabilizers in marine food web', *Science of the Total Environment*, 825.

Wick, Arne, Bjoern Jacobs, Uwe Kunkel, Peter Heininger, and Thomas A. Ternes. 2016. 'Benzotriazole UV stabilizers in sediments, suspended particulate matter and fish of German rivers: New insights into occurrence, time trends and persistency', *Environmental Pollution*, 212: 401-12.

Yao, Li, Jian-Liang Zhao, You-Sheng Liu, Yuan-Yuan Yang, Wang-Rong Liu, and Guang-Guo Ying. 2016. 'Simultaneous determination of 24 personal care products in fish muscle and liver tissues using QuEChERS extraction coupled with ultra pressure liquid chromatography-tandem mass spectrometry and gas chromatography-mass spectrometer analyses', *Analytical and Bioanalytical Chemistry*, 408: 8177-93.

Zhang, Zi-Yang, Yi-Heng Xu, Ke-Yan Chen, Mei-Hang Zhang, Chun-Yan Meng, Xue-Sheng Wang, and Man-Man Wang. 2023. 'Flower-like molybdenum disulfide/cobalt ferrite composite for the extraction of benzotriazole UV stabilizers in environmental samples', *Microchimica Acta*, 190.

Zhao, Ming-Liang, Yan Chen, Gui-Peng Yang, and Rong Chen. 2023. 'Simultaneous determination of benzothiazoles, benzotriazoles, and benzotriazole UV absorbers by solid-phase extraction-gas chromatography-mass spectrometry', *Environmental Science and Pollution Research*.

Zhao, Xue, Zi-Feng Zhang, Lei Xu, Li-Yan Liu, Wei-Wei Song, Fu-Jie Zhu, Yi-Fan Li, and Wan-Li Ma. 2017. 'Occurrence and fate of benzotriazoles UV filters in a typical residential wastewater treatment plant in Harbin, China', *Environmental Pollution*, 227: 215-22.
