# Supplementary material for: Neurotoxicity of Benzotriazole Ultraviolet Stabilizers in Teleost Fishes: A Review
Source: Toxics. 2024 Feb 2;12(2):125. doi: 10.3390/toxics12020125 (PMC10891865; doi:10.3390/toxics12020125)
Supplement: Supplementary file 1 [file toxics-12-00125-s001.zip › Table S2.pdf]

| Table S2. Occurrence of ultraviolet stabilizers in aquatic environments. | | | | |
|---|---|---|---|---|
| **Composition** | **Sample location** | **Sample type** | **Concentrations Median (range)** | **Reference** |
| **BUVSs** | Spain | seawater | 1095.39 ng/L (3.65-2998.51 ng/L） | [1-5] |
| | | Sewage | 184.93 ng/L (12.8-445.7 ng/L） | [3, 4, 6] |
| | | Groundwater | 1742.5 ng/L (ND-3290 ng/L) | [5] |
| | | Sediments | 12329.58 ng/g dw (10244.52-15158 ng/g dw) | [2] |
| | China | Seawater | 75.22 ng/L (10.73-281 ng/L） | [7-12] |
| | | Wastewater | 109.19 ng/L (39.61-235.54 ng/L) | [13] |
| | | Sediment | 6.91 ng/g dw (0.36-21.2 ng/g dw） | [10, 14, 15] |
| | Indian | Water | 617.20 ng/L (5.5-3037.8 ng/L） | [16-19] |
| | | Sediment | 3.5 ng/g dw (3.49-3.9 ng/g dw) | [16] |
| | | Sludge | 70.4 ng/g dw (38.8-102 ng/g dw) | [17] |
| | Portugal | water | 5.2 ng/L | [20] |
| | Greece | water | 580.15 ng/L | [21] |
| | South Korea | Seawater | 5.14ng/L (4.73-8.6 ng/L) | [22] |
| | | Sediment | 1.56ng/L (0.581-6.62 ng/L) | [22] |
| | Canada | water | 23.52 ng/L (0.452-86.35 ng/L) | [23-25] |
| | | Sediment | 9 ng/L (ND-43 ng/L) | [23] |
| | Norway | Sediment | 23.2 ng/L (37.2-63.2 ng/L) | [26] |
| | Switzerland | Surface water | 464 ng/L (11-917 ng/L) | [27] |
| | | Groundwater | 46.5 ng/L (16-77 ng/L) | [27] |
| | Tunisia | Seawater | 300.5 ng/L (47.1-1652 ng/L) | [28] |
| | | Sediments | 2082 ng/g dw (587-4252 ng/g dw) | [28] |

| | | | | |
|---|---|---|---|---|
| | Australia | Ground water | 645.4 ng/L | [29] |
| | Japan | Sediment | 15 ng/g dw (7.9-720 ng/g dw) | [30] |
| | German | Sediment | 50.08 ng/g dw | [31] |
| | Brazil | Sediment | 79.11 ng/g dw (ND-630 ng/g dw) | [32] |


1. Garcia-Guerra, R.B., et al., *Rapid monitoring of residual UV-stabilizers in seawater samples from beaches using fabric phase sorptive extraction and UHPLC-MS/MS.* Chemosphere, 2016. **164**: p. 201-207.
2. Montesdeoca-Esponda, S., et al., *Monitoring and environmental risk assessment of benzotriazole UV stabilizers in the sewage and coastal environment of Gran Canaria (Canary Islands, Spain).* Journal of Environmental Management, 2019. **233**: p. 567-575.
3. Montesdeoca-Esponda, S., et al., *Development of a sensitive determination method for benzotriazole UV stabilizers in enviromental water samples with stir bar sorption extraction and liquid desorption prior to ultra-high performance liquid chromatography with tandem mass spectrometry.* Journal of Separation Science, 2013. **36**(13): p. 2168-2175.
4. Montesdeoca-Esponda, S., Z. Sosa-Ferrera, and J. Juan Santana-Rodriguez, *On-line solid-phase extraction coupled to ultra-performance liquid chromatography with tandem mass spectrometry detection for the determination of benzotriazole UV stabilizers in coastal marine and wastewater samples.* Analytical and Bioanalytical Chemistry, 2012. **403**(3): p. 867-876.
5. Serra-Roig, M.P., et al., *Occurrence, fate and risk assessment of personal care products in river–groundwater interface.* Science of The Total Environment, 2016. **568**: p. 829-837.
6. Montesdeoca-Esponda, S., et al., *Fabric phase sorptive extraction followed by UHPLC-MS/MS for the analysis of benzotriazole UV stabilizers in sewage samples.* Analytical and Bioanalytical Chemistry, 2015. **407**(26): p. 8137-8150.
7. Pei, J., et al., *Occurrence, bioaccumulation and ecological risk of organic ultraviolet absorbers in multiple coastal and offshore coral communities of the South China Sea.* Science of the Total Environment, 2023. **868**.
8. Song, S., et al., *Occurrence and removal of benzotriazole ultraviolet stabilizers in a wastewater treatment plant in China.* Environmental Science-Processes & Impacts, 2014. **16**(5): p. 1076-1082.
9. Zhao, M.-L., et al., *Simultaneous determination of benzothiazoles, benzotriazoles, and benzotriazole UV absorbers by solid-phase extraction-gas chromatography-mass spectrometry.* Environmental Science and Pollution Research, 2023.

10. Tang, Z., et al., *Occurrence, distribution and ecological risk of ultraviolet absorbents in water and sediment from Lake Chaohu and its inflowing rivers, China.* Ecotoxicology and Environmental Safety, 2018. **164**: p. 540-547.
11. Hu, L.-X., et al., *Continuous input of organic ultraviolet filters and benzothiazoles threatens the surface water and sediment of two major rivers in the Pearl River Basin.* Science of the Total Environment, 2021. **798**.
12. Zhang, Z.-Y., et al., *Flower-like molybdenum disulfide/cobalt ferrite composite for the extraction of benzotriazole UV stabilizers in environmental samples.* Microchimica Acta, 2023. **190**(2).
13. Zhao, X., et al., *Occurrence and fate of benzotriazoles UV filters in a typical residential wastewater treatment plant in Harbin, China.* Environmental Pollution, 2017. **227**: p. 215-222.
14. Apel, C., J. Tang, and R. Ebinghaus, *Environmental occurrence and distribution of organic UV stabilizers and UV filters in the sediment of Chinese Bohai and Yellow Seas.* Environmental Pollution, 2018. **235**: p. 85-94.
15. Li, Y., et al., *Occurrence and distribution of organic ultraviolet absorbents in sediments from small urban rivers, Tianjin, China: Implications for risk management.* Ecotoxicology and Environmental Safety, 2022. **230**.
16. Vimalkumar, K., et al., *Occurrence of triclocarban and benzotriazole ultraviolet stabilizers in water, sediment, and fish from Indian rivers.* Science of the Total Environment, 2018. **625**: p. 1351-1360.
17. Karthikraj, R. and K. Kannan, *Mass loading and removal of benzotriazoles, benzothiazoles, benzophenones, and bisphenols in Indian sewage treatment plants.* Chemosphere, 2017. **181**: p. 216-223.
18. Vimalkumar, K., et al., *Screening of antimicrobials, fragrances, UV stabilizers, plasticizers and preservatives in sewage treatment plants (STPs) and their risk assessment in India.* Chemosphere, 2022. **308**.
19. Khare, A., et al., *Occurrence, spatio-temporal variation and ecological risk assessment of benzotriazole ultraviolet stabilizers (BUVs) in water and sediment of rivers in central India.* The Science of the total environment, 2023: p. 163381-163381.
20. Carpinteiro, I., et al., *Combining stir-bar sorptive extraction and large volume injection-gas chromatography-mass spectrometry for the determination of benzotriazole UV stabilizers in wastewater matrices.* Journal of Separation Science, 2012. **35**(3): p. 459-467.
21. Asimakopoulos, A.G., et al., *Occurrence and removal efficiencies of benzotriazoles and benzothiazoles in a wastewater treatment plant in Greece.* Science of the Total Environment, 2013. **452**: p. 163-171.
22. Wang, W., I.-S. Lee, and J.-E. Oh, *Specific-accumulation and trophic transfer of UV filters and stabilizers in marine food web.* Science of the Total Environment, 2022. **825**.

23. Dalpe Castilloux, A., et al., *Distribution and Fate of Ultraviolet Absorbents and IndustrialAntioxidants in the St. Lawrence River, Quebec, Canada.* Environmental Science & Technology, 2022. **56**(8): p. 5009-5019.
24. Lu, Z., et al., *Occurrence and fate of substituted diphenylamine antioxidants and benzotriazole UV stabilizers in various Canadian wastewater treatment processes.* Water Research, 2017. **124**: p. 158-166.
25. Lu, Z., et al., *Distribution, Partitioning and Bioaccumulation of Substituted Diphenylamine Antioxidants and Benzotriazole UV Stabilizers in an Urban Creek in Canada.* Environmental Science & Technology, 2016. **50**(17): p. 9089-9097.
26. Langford, K.H., et al., *Environmental occurrence and risk of organic UV filters and stabilizers in multiple matrices in Norway.* Environment International, 2015. **80**: p. 1-7.
27. Kahle, M., et al., *HYDROPHILIC ANTHROPOGENIC MARKERS FOR QUANTIFICATION OF WASTEWATER CONTAMINATION IN GROUND- AND SURFACE WATERS.* Environmental Toxicology and Chemistry, 2009. **28**(12): p. 2528-2536.
28. Fenni, F., et al., *Contaminants of emerging concern in marine areas: First evidence of UV filters and paraben preservatives in seawater and sediment on the eastern coast of Tunisia.* Environmental Pollution, 2022. **309**.
29. Liu, Y.-S., et al., *Simultaneous determination of benzotriazoles and ultraviolet filters in ground water, effluent and biosolid samples using gas chromatography-tandem mass spectrometry.* Journal of Chromatography A, 2011. **1218**(31): p. 5328-5335.
30. Nakata, H., S. Murata, and J. Filatreau, *Occurrence and Concentrations of Benzotriazole UV Stabilizers in Marine Organisms and Sediments from the Ariake Sea, Japan.* Environmental Science & Technology, 2009. **43**(18): p. 6920-6926.
31. Wick, A., et al., *Benzotriazole UV stabilizers in sediments, suspended particulate matter and fish of German rivers: New insights into occurrence, time trends and persistency.* Environmental Pollution, 2016. **212**: p. 401-412.
32. Mizukawa, A., et al., *Sediments as a sink for UV filters and benzotriazoles: the case study of Upper Iguacu watershed, Curitiba (Brazil).* Environmental Science and Pollution Research, 2017. **24**(22): p. 18284-18294.
