# Supplementary material for: Neurotoxicity of Benzotriazole Ultraviolet Stabilizers in Teleost Fishes: A Review
Source: Toxics. 2024 Feb 2;12(2):125. doi: 10.3390/toxics12020125 (PMC10891865; doi:10.3390/toxics12020125)
Supplement: Supplementary file 1 [file toxics-12-00125-s001.zip › Table S3.pdf]

Table S3: presents data for marine invertebrates

Reference	Chemicals	Dose	Tested organisms	Life stage	Exposure period	Endpoint	Results
[1]	UV-234,UV-326,UV-329,UV-P	0.1,1,10μg/L	*Corbicula fluminea*	Adult	21d	Filtering rate; Ach content; AchE activity	Filtering rate were increased, and ACh content as well as AChE activity were increased in C. fluminea from all BUVSs groups.
[2]	benzophenone-3 (BP3); 3-(4-methylbenzylidene)camph(4-MBC); octocrylene (OC)	0.09,1.12,14.13mg/kg	*Chironomus riparius*	larvae	2d	AChE activity	C. riparius larvae exposed to 0.09 mg/kg 4-MBC showed a decrease in AChE activity

1. Zhang, J., et al., *Comparative toxicogenomics of benzotriazole ultraviolet stabilizers at environmental concentrations in Asian clam<i> (Corbicula</i><i> fluminea):</i> Insight into molecular networks and behavior.* Journal of Hazardous Materials, 2023. **447**.
2. Campos, D., et al., *Toxicity of organic UV-filters to the aquatic midge <i>Chironomus riparius</i>.* Ecotoxicology and Environmental Safety, 2017. **143**: p. 210-216.

Studies have shown that benzotriazole cause significant changes in the expression levels of VTG, CYP1A1, and CYP19a in *Oryzias melastigma* at environmentally relevant concentrations (0.01 mg/L) detected in the aquatic environment[3]. The EC50 value of BT for Phaeodactylum tricornutum is 41mg/L[4]. UV-327 was exposed to sea urchin larvae for 4 days, and the growth was significantly inhibited at the level of 5 mg/L[5]. Toxicities of BP-1 , BP-3 , BP-4 and BP-8 to larvae and adults of two coral species, Pocillopora damicornis and Seriatopora caliendrum, were assessed at concentrations ranging from 0.1 – 1000 μ g/L. The EC50 value of BP-1 for larva of

Seriatopora caliendrum De-settlement is 184.13иg/L, BP-3 at 1000 иg/L can cause 95% of the larvae of Seriatopora caliendrum bleached and 50% of the nubbin corals bleached. BP-8 at 10 иg/L results in 83.3% Seriatopora caliendrum polyps were retracted, and BP-8 at 1000 иg/L results in 100% Pocillopora damicornis corals polyps were retracted[6].

1. Zhang, J., et al., *Comparative toxicogenomics of benzotriazole ultraviolet stabilizers at environmental concentrations in Asian clam<i> (Corbicula</i><i> fluminea):</i> Insight into molecular networks and behavior.* Journal of Hazardous Materials, 2023. **447**.
2. Campos, D., et al., *Toxicity of organic UV-filters to the aquatic midge <i>Chironomus riparius</i>.* Ecotoxicology and Environmental Safety, 2017. **143**: p. 210-216.
3. He, T.T., et al., *Estrogenic potential of benzotriazole on marine medaka (<i>Oryzias melastigma</i>).* Ecotoxicology and Environmental Safety, 2012. **80**: p. 327-332.
4. Canova, L., et al., *A Comparative Test on the Sensitivity of Freshwater and Marine Microalgae to Benzo-Sulfonamides, -Thiazoles and -Triazoles.* Applied Sciences-Basel, 2021. **11**(17).
5. Shore, E.A., et al., *Four plastic additives reduce larval growth and survival in the sea urchin Strongylocentrotus purpuratus.* Marine Pollution Bulletin, 2022. **175**.
6. He, T.T., et al., *Comparative toxicities of four benzophenone ultraviolet filters to two life stages of two coral species.* Science of the Total Environment, 2019. **651**: p. 2391-2399.
